# Long-term neurologic and cardiac correction by intrathecal gene therapy in Pompe disease

**DOI:** 10.1186/s40478-017-0464-2

**Published:** 2017-09-06

**Authors:** J. Hordeaux, L. Dubreil, C. Robveille, J. Deniaud, Q. Pascal, B. Dequéant, J. Pailloux, L. Lagalice, M. Ledevin, C. Babarit, P. Costiou, F. Jamme, M. Fusellier, Y. Mallem, C. Ciron, C. Huchet, C. Caillaud, M-A Colle

**Affiliations:** 1 INRA UMR U703, Animal Pathophysiology and Biotherapy for Muscle and Nervous system Diseases, UMR 703 PAnTher INRA/ONIRIS, ONIRIS, CS 40706, F-44307 Nantes Cedex 03, France; 2 LUNAM Université, Oniris, Nantes-Atlantic National College of Veterinary Medicine, Food Science and Engineering, CS 44706, F-44307 Nantes, France; 3 LUNAM Université, Université de Nantes, UFR Sciences et Techniques, F-44322 Nantes, France; 4 SOLEIL French National Synchrotron Facility, Saint-Aubin, Gif-sur Yvette, F-91192 Paris, France; 5 LUNAM université, Oniris, Department of Medical Imaging, Centre de Recherche et d’Investigation Préclinique, F-44307 Nantes, France; 6 LUNAM université, Oniris, Physiopathologie Animale et Pharmacologie Fonctionnelle, F-44307 Nantes, France; 7 INSERM UMR1087/CNRS UMR6291, l’Institut du Thorax, F-44322 Nantes, France; 8 INSERM UMR1151/CNRS UMR8253, Institut Necker Enfants Malades, F-75993 Paris, France; 9 Université Paris Descartes, F-75006 Paris, France

**Keywords:** Pompe disease, Gene therapy, CNS, Intra-cerebrospinal fluid injection

## Abstract

Pompe disease is a lysosomal storage disorder caused by acid-α-glucosidase (GAA) deficiency, leading to glycogen storage. The disease manifests as a fatal cardiomyopathy in infantile form. Enzyme replacement therapy (ERT) has recently prolonged the lifespan of these patients, revealing a new natural history. The neurologic phenotype and the persistence of selective muscular weakness in some patients could be attributed to the central nervous system (CNS) storage uncorrected by ERT. GAA-KO 6neo/6neo mice were treated with a single intrathecal administration of adeno-associated recombinant vector (AAV) mediated gene transfer of human GAA at 1 month and their neurologic, neuromuscular, and cardiac function was assessed for 1 year. We demonstrate a significant functional neurologic correction in treated animals from 4 months onward, a neuromuscular improvement from 9 months onward, and a correction of the hypertrophic cardiomyopathy at 12 months. The regions most affected by the disease i.e. the brainstem, spinal cord, and the left cardiac ventricular wall all show enzymatic, biochemical and histological correction. Muscle glycogen storage is not affected by the treatment, thus suggesting that the restoration of muscle functionality is directly related to the CNS correction. This unprecedented global and long-term CNS and cardiac cure offer new perspectives for the management of patients.

**Electronic supplementary material:**

The online version of this article (doi:10.1186/s40478-017-0464-2) contains supplementary material, which is available to authorized users.

## Introduction

Pompe disease, also known as type II glycogenosis, is a lysosomal storage disease (LSD) caused by mutation in the acid-α-glucosidase (GAA) gene. In classic infantile Pompe disease, the severe GAA activity loss causes multi-system and early-onset glycogen storage, especially within the heart and muscles, and early death from cardiorespiratory failure [7]. Infantile Pompe disease is also characterized by marked glycogen storage within neurons and glial cells, and also reactive astrocytosis and hypomyelination [12, 18, 39, 40, 61, 62]. Involvement of the central nervous system (CNS) has recently regain interest due to the emergence of a new neurologic phenotype in some patients under enzyme replacement therapy (ERT) [5, 14, 48, 67]. Patients, who live longer due to cardiac correction, reveal a new natural history and raise questions about the pathophysiology of the disease. Specifically, the emergent neurologic phenotype in some patients and the frequent persistence of bulbar muscular weakness could be attributed to CNS lesions, uncorrected by ERT because of the blood-brain-barrier [34, 48, 57]. In infantile Pompe disease patients, the glycogen storage diffusely affects brainstem motor and sensory neurons, and the whole spinal cord sensory neurons, interneurons, and motor neurons [39]. Recently, a genomic CNS screening in a Pompe mouse model confirms that systemic absence of GAA induces a complex neuropathological cascade in the spinal cord [64]. Furthermore, the weak correction of some group of skeletal muscles by ERT could be due to the persistence of storage in motor neurons in addition to others factors such as the low uptake of recombinant GAA (rGAA) in muscles associated with paucity of the cation-independent-mannose-6-phosphate receptor (CI-MPR) and abnormal receptor trafficking [9, 35, 36, 45], and the apparition of anti-rGAA antibodies in treated patients [1, 2, 16, 66]. Recently, the specific implication of phrenic motor neurons in the pathophysiology of the respiratory failure has been demonstrated in a mouse model of Pompe disease [18, 23, 37, 44, 65]. These results suggest that a global cardiac, muscular, and CNS targeting therapy is needed to fully reverse the phenotype of infantile Pompe disease. Gene therapy is currently the most promising approach to target durably both peripheral organs and CNS [6]. In particular, strategies that diffusely target the CNS are necessary to address the lysosomal pathology.

Extensive reporter gene transfer to the CNS has been achieved by we and others after intrathecal injection, i.e. delivery into the cerebrospinal fluid, of recombinant Adeno-Associated-Vectors (AAV) serotype 9 and rh10 [3, 4, 25, 27, 32, 41, 49, 69]. Moreover, this strategy has proven efficient for the treatment of lysosomal storage diseases [22, 28, 70] and more recently of motor neuron diseases [41–43, 69]. As a proof-of-concept we assessed the efficacy of a single intrathecal delivery of AAVrh10 or AAV9 vectors expressing GAA on the neurological and neuromuscular function in the 6neo/6neo murine model that recapitulates the pathology of the disease [46, 54]. Serotype rh10 is already employed in clinical trials of several neurological diseases (Sanfilippo type A NCT01474343, Metachromatic Leukodystrophy NCT01801709, Batten disease NCT01161576 and NCT01414985 on ClinicalTrials.gov) with good safety reports. Serotype 9 is known to have a robust motor neuron tropism in several large animal species including non human primates [4, 25, 27, 41].

Our results show that a single intrathecal delivery of AAVrh10- or AAV9-CAG-hGAA to 1 month old 6neo/6neo mice enable significant and sustained neurologic and neuromuscular correction for 1 year that correlates with CNS lysosomal pathology reversion. Treatments lead to partial restoration of the muscular strength despite unmodified muscle glycogen storage, thus suggesting that the global neuromuscular amelioration is directly and only related to the CNS rescue. Lastly, our data show for the first time that in addition to CNS correction, the serotype 9 restores GAA levels in the heart and alleviates the cardiac storage and the hypertrophic cardiomyopathy.

## Materials and methods

### Study design

#### Experimental design

**Fig. 1:**
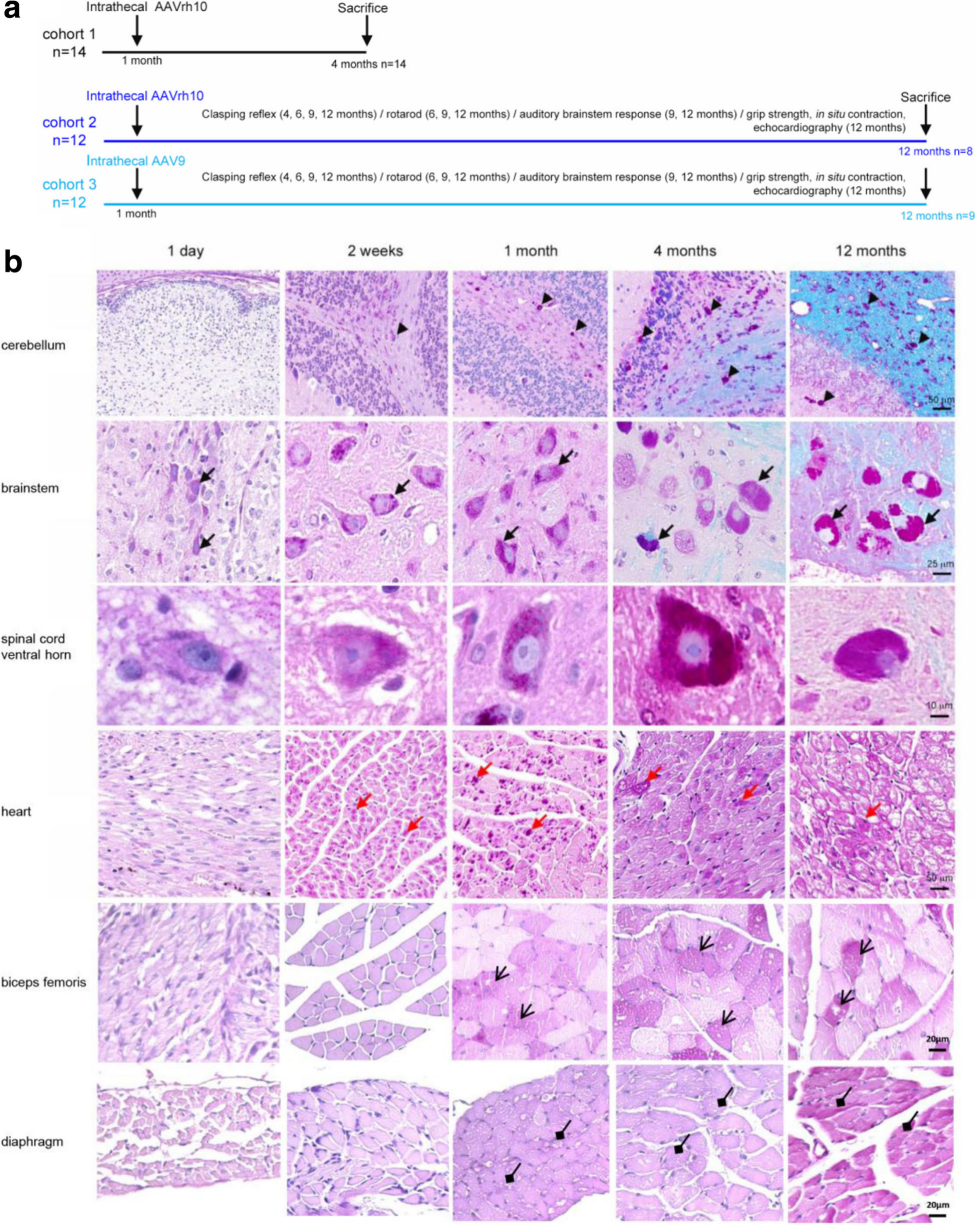
Experimental design and natural history of the disease in 6neo/6neo mice. **a** Overview of the experimental design. **b** Kinetic of apparition of the glycogen storage in Pompe mice. Paraffin-embedded sections, PAS-luxol (CNS) or PAS (heart, biceps femoris, and diaphragm) stain. Black arrowheads point to glycogen storage in glial cells, black arrows to storage in motor neurons, and red arrows to storage in cardiomyocytes. Glycogen (purple) is present from birth in the motor neurons of the brainstem, and from 2 weeks in the motor neurons of the spinal cord, in the glial cells of the cerebellum, and in the heart, and from 1 month for biceps femoris and diaphragm

This was an observational preclinical study designed to search for possible differences among experimental treatment groups (gene therapy by AAV9-CAG-hGAA or AAVrh10-CAG-hGAA, mock-treatment). Treatment effect was assessed in vivo by functional neurologic, neuromuscular, and cardiac testing. Two endpoints were selected, a short-term (4 months) and a long-term (12 months), to sample and analyze the organs of the animals. The experimental design is outlined on Fig. 1a.

### Randomization and blinding

This was an open-label non-randomized study. Seven investigators blinded to the animal’s identity performed functional (JH, QP), histological (JH, BD), cardiac (MF), electrophysiological (PC) and molecular biology (CB, CC) analyzes independently.

### Predefined study components

Preliminary data obtained by following the natural history of the disease in the murine model indicated that eight animals were required in each group to detect a 20% difference in muscle grip strength with 80% power and an alpha of 0.05 while six animals were required to detect a 10% difference in brainstem auditory response (BAR) interpeak latency P1-P5 with 80% power and an alpha of 0.05. As we anticipated natural mortality in the long-term 12-month study, we decided to inject a minimum of 11 animals per group.

### Sample size

According to the power analysis and to the availability of animals when the long-term study was initiated, fifteen wild-type (WT) animals were mock-treated, eleven 6neo/6neo Pompe mice were mock-treated, and twelve 6neo/6neo Pompe mice were injected with AAVrh10-CAG-hGAA or AAV9-CAG-hGAA. At the end of the twelve-month study, fourteen WT animals, nine mock Pompe mice, eight AAVrh10 and nine AAV9 Pompe mice were alive. The causes of natural death are listed in supplementary materials and methods (Additional file 1: Table S1); all animals were necropsied by a European College of Veterinary Pathologists certified veterinary pathologist (JH). A short-term four-month study was also performed with ten WT mock-treated mice and fourteen mock-treated or AAVrh10-treated Pompe mice. No natural death occurred during the short-term study.

### Selection of start and endpoints

Preliminary data obtained by following the natural history of the disease in the murine model indicated that motor neuron storage is minimal the day of birth, slight at 2 weeks, moderate at 1 month, marked at 4 months and severe at 12 months (Fig. 1b). Mice are asymptomatic at 1 month, and display a progressive aggravation of neurological and neuromuscular functional tests from 4 months onwards. We chose to inject the treatment at the age of 1 month (well developed lesions, presymptomatic stage) and performed two endpoints: 4 months (for AAVrh10-CAG-hGAA), and 12 months (AAVrh10 and AAV9-CAG-hGAA). We added this long-term group injected with AAV9-CAG-hGAA because we demonstrated at the same time in our laboratory that AAV9 was more efficient than AAVrh10 after intrathecal injection for the targeting of motor neurons in the non-human primate (B. Joussemet et al. ESGCT conference, Madrid 2013). With a translational objective in mind, we therefore initiated the long-term study with an additional AAV9-CAG-hGAA group.

### Animals

Breeding heterozygous 6neo/6neo mice were kindly provided by Nina Raben (NIH, Bethesda USA) and housed under specific pathogen free conditions in the accredited rodent facility of Oniris (Nantes-Atlantic National College of Veterinary Medicine, Food Science and Engineering, Nantes, France). Mice were genotyped as previously described [46] and homozygous breeding was performed after the first heterozygous generation. WT littermates were bred as controls. Males only were used in order to limit the hormone-related variations for the functional tests. Experiments were carried out according to European guidelines for the care and use of experimental animals, and were reviewed and approved by the regional ethics committee (CEEA Pays de la Loire, authorization number CEEA-2012-210).

### Construction and production of AAV-GAA vectors

Human acid-α-glucosidase complementary DNA (NCBI reference NM_001079804) was cloned under the control of CAG i.e. the cytomegalovirus early enhancer element and chicken beta-actin promoter. Plasmid’s functionality was verified in vitro by transfection into HEK293 cells and by a secretion-uptake assay performed on Pompe patients fibroblasts kindly provided by C. Caillaud (Institut Necker Enfants Malades, Paris) as described on Additional file 1: Figure S1. Pseudotyped AAVrh10 and AAV9 vectors were generated by packaging AAV2-based recombinant genome into serotype rh10 or 9 capsids, as previously described [47]. Briefly, the vectors were produced by helper virus-free co-transfection in HEK293 cells, using (i) the adenovirus helper and AAV packaging plasmid encoding the adenoviral genes together with the rep2 and cap9 or cap10 genes and (ii) the AAV2 plasmid containing the CAG-hGAA genome. Recombinant vectors were purified by double-CsCl ultracentrifugation followed by dialysis against Dulbecco’s phosphate-buffered saline (PBS) with calcium chloride and magnesium chloride. Vector titres, expressed as viral genomes per millilitre (vg/ml), were 6.4 × 10^12^ vg/ml and 4.8 × 10^12^ vg/ml for AAVrh10-CAG-hGAA; 1.0 × 10^13^ vg/ml and 1.3 × 10^13^ vg/ml for AAV9-CAG-hGAA as measured by dot blot hybridization and real-time polymerase chain reaction (PCR) respectively.

### Administration of vectors

One-month-old mice were anaesthetized with ketamine 100 mg/kg (Imalgene®, Merial) and xylazine 10 mg/kg (Rompun®, Bayer) and received 10^11^ vg (5 × 10^12^ vg/kg) of either AAVrh10 or AAV9 or PBS in a total volume of 10 μl infused in the *cisterna magna* under the control of an operating microscope and at a controlled rate of 1 μl per minute.

### Viral particles detection in the blood

To study the kinetic of rAAV distribution from the CSF to the blood compartment, 4 WT mice were injected in the *cisterna magna* with 10^11^ vg of AAVrh10-CAG-GFP and 4 with AAV9-CAG-GFP. Vectors were titer-matched and injected in the same volume (10 μl). Sera were collected 1 h, 2 days, and 1, 2, 4, 6, and 8 weeks after the injection. Viral DNA was extracted using Nucleospin RNA virus (Macherey-Nagel) and AAV particles were quantified by qPCR (polyA SV40 qPCR assay).

### GAA activity and glycogen storage quantification

For GAA activity assays, the CNS, heart, liver, and skeletal muscles were rapidly dissected after euthanasia and PBS perfusion. Brains were sectioned into four coronal slabs of ~2 mm thickness and the spinal cord into two coronal slabs. All tissues were then snap-frozen in liquid nitrogen, and stored at −80 °C until biochemical analyses were performed. Tissues were homogenized in a phosphate buffer, homogenates were centrifuged at 13,000 rpm for 10 min at 4 °C and the resulting supernatant was assayed for GAA activity by measuring cleavage of 4-methylumbelliferyl-α-D-glucopyranoside aſter incubation for 1 h at 37 °C as previously described [46]. Protein concentration was measured using Bicinchoninic Acid method per manufacturer’s instructions (B9643, Sigma-Aldrich). Biochemical measurement of glycogen content was then performed as described elsewhere [20]. Tissue extracts were boiled for 3 min and incubated at 54 °C for 1 h in the presence or absence of *Aspergillus niger* amylo-α-1,4-α-1,6 glucosidase (5 U/ml; Roche, Mannheim, Germany) which converts glycogen to glucose. Samples were centrifuged and glucose level was determined in the supernatant using Glucose RTU kit (Biomerieux, Lyon, France) per manufacturer’s instructions.

### GAA immunoblot analyses (WB and ELISA)

A rabbit and a rat anti-hGAA polyclonal antibody were produced in our laboratory by subcutaneous immunisation with recombinant human GAA (rGAA, Myozyme®, Genzyme) in complete Freund adjuvant followed by boosters in incomplete Freund adjuvant. After serum immunoglobulin’s purification (Ig-Adem kit, Ademtech), the specificity of our antibodies was checked by western blot analysis by detection of rGAA at 110 kD. The proteins in tissue extracts were separated by SDS-PAGE gel electrophoresis, and the rat purified antibody was used to blot GAA in the organs of AAV-treated Pompe mice and PBS-treated mice as negative controls. Detection was performed with a secondary anti-rat antibody coupled to AlexaFluor®680 (Life Technologies) and the Odyssey infrared imaging system (LI-COR Biotechnology Inc.). For sandwich ELISA, plates were coated with purified rabbit anti-GAA antibodies, tissue extracts were incubated, and rat anti-GAA antibodies revealed GAA. Horseradish peroxidase (HRP) conjugated donkey anti-rat IgG (1:5000, r712–035-150; Jackson Immuno Research) followed by Streptavidin/HRP (1:1000, P0397; DakoCytomation) was added and 3,3′,5,5′- Tetramethylbenzidine (TMB, BD Biosciences) was used as substrate. Reactions were stopped with 2 N H2SO4 and reading was determined at 450 nm. Quantification was done using serial dilutions of rGAA (Myozyme) as standard.

### Anti-GAA antibody detection

An indirect ELISA was used to detect GAA specific antibodies. Plates were coated with rGAA (Myozyme, Genzyme Corporation) overnight, rinsed, and blocked with 5% milk in PBS. Samples in serial dilutions, controls, and blanks were added and incubated for 2 h at 37 °C. HRP conjugated donkey anti-rat IgG (1:5000, r712–035-150; Jackson Immuno Research) followed by Streptavidin/HRP (1:1000, P 0397; DakoCytomation) was added and 3,3′,5,5′- Tetramethylbenzidine (TMB, BD Biosciences) was used as substrate. Reactions were stopped with 2 N H2SO4 and reading was determined at 450 nm. Positivity was determined with control sera collected prior to the injections using a cut-off value of 0.200 OD.

### Vector genome quantification

Vector genome copy numbers per diploïd genome were quantified from 100 ng and 50 ng of total DNA extracted with phenol/chloroform method. Sequences from the transgene (hGAA) and the endogenous gene (mouse albumin) were amplified by the probe-based real time PCR method (Promega, Wisconsin, USA) on a Thermocycler CFX96 (Biorad, California, USA) using the following cycling conditions: 5 min at 95 °C, then 15 s at 95 °C and 1 min at 60 °C for 40 cycles. Each sample was analyzed in triplicate. The n-fold differences in the hGAA transgene copy number relative to the *albumin* gene was determined using the 2^∆Ct^ method, where the ∆Ct corresponded to the subtraction of the Ct value of the hGAA sequence from the mean Ct value of the *albumin* gene. Final results were expressed as vector copy number per 2n genome.

Primers and probes were designed using Oligo Primer Analysis Software v. 7 (Molecular Biology Insights Inc., Cascade, USA) and synthesized by Eurofins MWG Operon (Ebersberg, Germany). Primers and probe used for murine albumin amplification (used as internal control) were as follows: Forward: 5′- ACATAGCTTGCTTCAGAACGGT; Reverse: 5′-AGTGTCTTCATCCTGCCCTAAA;Probe:5’ATCATAGTATCCTAGTCCACAGGTTCTGCAGCACT. Primers and probe used for hGAA amplification were as follows: Formard: 5′- TTCGGCTTCTGGCGTGTG; Reverse: 5′-AGGAGCCGGTGGGAGCAG; Probe: 5′- AGAGCCTCTGCTAACCATGTTCATGCCTT.

### mRNA transcript analysis

Cardiac structural protein transcripts, myosin beta heavy chain 7 (myh7), actin alpha cardiac muscle 1 (actc1) and actin alpha 1 (acta1) were quantified using Quiagen Quantitect reverse transcription kit; 18 s ribosomal RNA was used as internal control.

Total RNA was isolated with an RNAeasy Mini Kit (Qiagen). cDNA was prepared using a Go Script™ Reserve Transcription System (Promega). Briefly, total RNA (100 ng) was reverse transcribed in a final volume of 20 μl with OligodT primers at 37 °C for 1 h according to the manufacturer’s instructions. The expression level of myh7, actc1, and acta1 were measured by RT-QPCR using SybrGreen assays. 18S ribosomal RNA was used as endogenous control. We used Quantitect Pimer Assays (Qiagen) to quantify expression of these three genes. Each assay was run in duplicate, with the Quantitect Sybr Green PCR Kit (Qiagen) on a thermocycler CFX96 (Biorad, California, USA) using the following cycling conditions: 15 min at 95 °C, then 15 s at 95 °C and 30 s at 55 °C for 40 cycles.

Each replicate cycle threshold (Ct) was normalized to the Ct of the endogenous control on a per sample basis. The comparative Ct method was used to calculate relative levels of myh7, actc1 and acta1 expression.

### Histological and immunofluorescence analyses

#### Tissue processing

For histological analyses, the mice were perfused with paraformaldehyde (PFA) 4% in PBS; organs were rapidly dissected (brain, spinal cord, dorsal root ganglia, nerves, heart, liver, and muscles) and the CNS tissues were post-fixed in PFA 4% periodic acid 1% as described elsewhere [54]. All organs were trimmed; half was embedded in paraffin and the remaining was frozen in mounting medium (Cryomount, HistoLab) after 6% and 30% sucrose cryoprotection. In one animal of each group, 2x1x1 mm portions of the cervical spinal cord and the heart were fixed with 2.5% glutaraldehyde in a 0.1 M phosphate buffer at pH 7.4 for 24 h at 4 °C, rinsed with 0.1 M phosphate buffer and post-fixed with 1% osmium tetroxide (OsO4). Tissues were dehydrated through a graded aqueous ethanol series and embedded in Epon resin (Epoxy Embedding Medium Kit, 45,359-1EAF Sigma Aldrich).

### Histology

Paraffin-embedded 10 μm serial sections were stained with hemalun-eosin-saffron (HES), periodic-acid-Schiff (PAS), or PAS luxol-fast blue and observed using the Nikon Eclipse 80i® microscope and NIS-element software. Quantitative assessment of the glycogen storage in the spinal cord ventral horns (percentage of PAS positive motor neurons) was done using the Fiji freeware. Briefly, 3 sections of cervical spinal cord and 3 sections of lumbar spinal cord each separated by 1 mm-thick to avoid double-counting were analyzed in 4 animals per group. Motor neurons, *ie* large neurons located in the lamina IX of Rexed were pointed and PAS positive motor neurons were counted. A mean total of 123.5 (±18.2) and 121.3 (±14.2) motor neurons per animal were counted in the cervical and lumbar segments respectively. There was no difference in the total number of MN between groups. For transmission electron microscopy, ultra-thin sections (80 nm) were obtained using an ultramicrotome (UC7, Leica) and collected on copper grids then contrasted with uranyl acetate 2% in aqueous solution. Sections were examined using an electron transmission micrograph (JEM-1230, Jeol) with an accelerating voltage of 80 kV.

### Immunohistochemistry

Frozen 20 μm cryosections were used to investigate the cellular localization of GAA in the CNS. Immunofluorescence colocalization study was performed with the following primary antibodies: rabbit or rat polyclonal anti-GAA antibody, mouse monoclonal anti-neuN for neurons (Chemicon, MAB377, 1/800), rabbit polyclonal anti-olig2 for oligodendrocytes (Chemicon, Ab9610, 1/500), rabbit polyclonal anti-Iba1 (*Ionized calcium-binding adaptor molecule 1*) for microglial cells (Wako 019–19,741, 1/1000), and rabbit polyclonal anti-GFAP *(Glial Fibrillary Acidic Protein)* for astrocytes (Dako® Z0334, 1/5000). Briefly, primary antibodies were incubated overnight at 4 °C after permeabilization and the secondary antibodies labelled with Alexa® red 555 or green 488 (Life Technologies; 488A21121–1/300, 555A21429–1/500, 488A11008–1/500, 555A21434–1/500) were incubated for 1 h at room temperature. Confocal laser scanning microscopy was performed with a mDigital Eclipse C1 (Nikon) and a LSM 780 (Zeiss). Reactive astrocytosis was quantitatively expressed as the percentage of GFAP positive area using Fiji freeware in cervical spinal cord sections. Briefly, a region of interest (ROI) was defined in the ventral horns (encompassing the laminae IX of Rexed) and another one in the dorsal white matter tracts and a binary was done to discriminate the red PAS positive surface from the rest of the area. The area of PAS positive surface relative to the total surface of the ROI was automatically calculated.

### Fourier transform infrared (FT-IR) microspectroscopy

Cervical spinal cord samples from three AAVrh10 treated, three mock-treated, and three WT animals were studied. Synchrotron FT-IR microspectroscopic analysis was performed at the SOLEIL synchrotron (Gif/Yvette, France) using an infrared microscope (Nicolet iN10, Thermo Scientific, USA) to collect prior chemical information at medium resolution in order to perform an Infra-Red mapping of the whole spinal cord section. Spectra were acquired in reflectance mode with an aperture of 25 μm and an acquisition step of 25 μm. Glycogen concentrations were determined by measure of the area under the curve for the glycogen peak at 1080 cm^−1^ normalized versus the protein peak area at 1654 cm^−1^ by using Omnic software (Thermoscientific). Relative concentration of glycogen was represented relative to amide bands of the proteins. High spatial resolution infrared spectral maps were then collected using the SMIS beamline (Spectroscopy and Microscopy in the infrared using synchrotron SOLEIL, Gif/Yvette, France). Formalin fixed paraffin embedded sections of spinal cord (8 μm) were placed on Zinc Sulfate windows (ZnS, 13 mm in diameter). Paraffin was removed from the tissue sections prior to FT-IR analysis. All spectra were recorded in transmission mode on a Continuum XL microscope (Thermo Scientific). The microscope comprises a motorized sample stage and a liquid nitrogen cooled mercury cadmium telluride (MCT-A) detector (50 μm element size). The microscope operates in confocal mode using a 32× infinity corrected Schwarzschild objective (NA = 0.65) and a matching 32× condenser. All spectra were recorded using a dual mask aperture of 10 × 10 μm^2^. Individual spectra were saved in log(1/R) format at 8 cm^−1^ spectral resolution, with 128 co-added scans encompassing the mid-IR region from 4000 to 800 cm^−1^. All infrared spectra were pre-processed and submitted to multivariate data analysis (The Unscrambler, CAMO Process AS, www.camo.com). Spectral data were first baseline corrected and unit vector normalized. Second derivatives of the spectral data were assessed (9-point Savitzky–Golay filter) to enhance the spectral resolution of the absorption bands. The second derivative infrared spectra pre-multiplied by −1 were analysed by applying principal component analysis. The computation of principal components was based on the non-linear iterative projections by alternating least-squares (NIPALS) algorithm. While the score plots allowed a comparison of the infrared spectra, the corresponding loading plots revealed the main characteristic absorption bands.

### Functional testing

The in vivo tests were blindly performed in the same sequence for each mouse, with equivalent time of rests in-between, and at the same time of the day.

### Neurological function

Clasping reflex was scored at 4, 6, 9, and 12 months of age; auditory brainstem response (ABR) was recorded at 9 and 12 months and rotarod testing was performed at 6, 9, and 12 months in PBS versus treated mice compared to B6;129 PBS injected WT mice. The hindleg clasping reflex assesses the inhibitory function of the CNS while a mice is suspended by its tail [40]. Mice with neurological impairment show abnormal reflex retraction of the legs and paws. A score of zero corresponded to normal placement, one to inconsistent retraction of one leg, two to permanent retraction of one leg, three to inconsistent retraction of both legs and four to permanent retraction of both legs. Auditory brainstem response was collected as described elsewhere [72] with a Medtronic instrument and keypoint software (Medtronic 2003, Keypoint 5.09). The stimulus modality was the alternative click with duration of 50 μsec and a frequence of 31 Hz. Auditory responses were recorded through disposable scalp needle electrodes 30G (Alpine Biomed, Skovlunde, Denmark). A veterinary electrophysiologist (PC) determined wave position. The wave I-V inter-peak latency represents the time required for neural impulses to conduct through the auditory brainstem, thus representing an objective and specific follow-up of the brainstem function in mice. Motor coordination and balance were evaluated with an accelerating rotarod recording the latency to fall when mice were submitted to an acceleration of 4 to 40 rpm over 5 and then 3 min. Mice were tested three times with rest periods of at least 1 min between measurements; the best performance was kept and used for statistical analysis.

### Neuromuscular function

Neuromuscular function was blindly assessed by the wire hang test at 4, 6, 9, and 12 months of age and the grip test at 4 and 12 months of age. For the wire hang test, the ability to hang upside down from a wire screen placed 50 cm above a large housing cage was measured as a latency to fall into the cage. Maximum time was 60 s; each mouse was tested five times, the minimal and the maximal score were excluded and the three remaining values were averaged. Grip test was performed as previously described [10]. Briefly, mice were placed with their four paws on a grid and were gently pulled backward until they released their grip. A grip meter (Bio-GT3, Bioseb, France), attached to a force transducer, measured the peak force generated. The mean result of three assays was normalized to the body weight.

### Contractile properties of fast- and slow-twitch muscles

*Extensor digitorum longus* (edl) and *soleus* muscles were prepared and analysed as described previously [10]. Briefly, twitch parameters and tetanic forces were recorded, normalized to the fresh muscle weight and analysed with Chart v4.2.3 (PowerLab 4/25 ADInstrument, Phymep, France).

### Cardiac function

Echocardiography was blindly performed at 12 months with the MyLab70 XVG device (Esaote, Indianapolis, IN) using a linear 18MHhz transducer. Probe selection and frequency, depth of field, overall gain, time-gain compensation, and focal zone were adjusted at the discretion of the sonographer to optimize image quality. Examination was performed on 2% isoflurane-anaesthetized mice. Measurements were realized after stabilization of the cardiac frequency between 350 and 380 beats/min. Images of the short axis were obtained in M-Mode. Left ventricular diameters were measured at end of diastole and end of systole as well as thickness of left ventricular posterior wall and interventricular septum in diastole.

### Statistical analysis

Data are expressed as average ± standard error of the mean (SEM). In order to take into account the repeated measurements design, differences between groups and interactions with time have been evaluated using linear mixed effects models (R Development Core Team 2011 R Foundation for Statistical Computing,Vienna, Austria. ISBN 3–900,051–07-0, URL http://www.R-project.org/). One-way ANOVA with post-hoc test Newman-Keuls was performed using XLStat software. Graphs and correlations between continuous variables were tested with the Pearson’s test (GraphPad software Prism 5.0). A *p*-value of 0.05 or less was considered significant.

## Results

### Intrathecal AAV-hGAA rapidly reverses the CNS pathology in the short term

One month-old Pompe mice received a single intrathecal injection of AAVrh10-CAG-hGAA at the dose of 5 × 10^12^ vg/kg and were sacrificed 3 months after the injection (*n* = 14; Fig. 1). We verified prior to the in vivo experiments that the recombinant GAA coded by our plasmid was correctly processed, secreted and endocytosed in vitro by using infantile Pompe disease fibroblasts grown in the presence of medium from CAG-hgaa transfected HEK293 cells (Additional file 1: Figure S1). We show at 4 months that the glycogen storage is cleared in the whole CNS of all treated mice, which were indistinguishable from mock-treated wild-type (WT) mice (Additional file 1: Figure S2a, b). Three months after injection, less than 15% of motor neurons in proximal spinal cord were reactive to periodic acid schiff (PAS), which stains intracellular accumulation of glycogen. In contrast, mock-treated Pompe mice had large motor neurons with massive accumulation of undegraded material. GAA activity was measured in both brain and spinal cord of treated mice and was closed to physiological values (Additional file 1: Figure S2c).

### Intrathecal AAV-hGAA prevents the development of neurological manifestations and sustains long-term correction

**Fig. 2:**
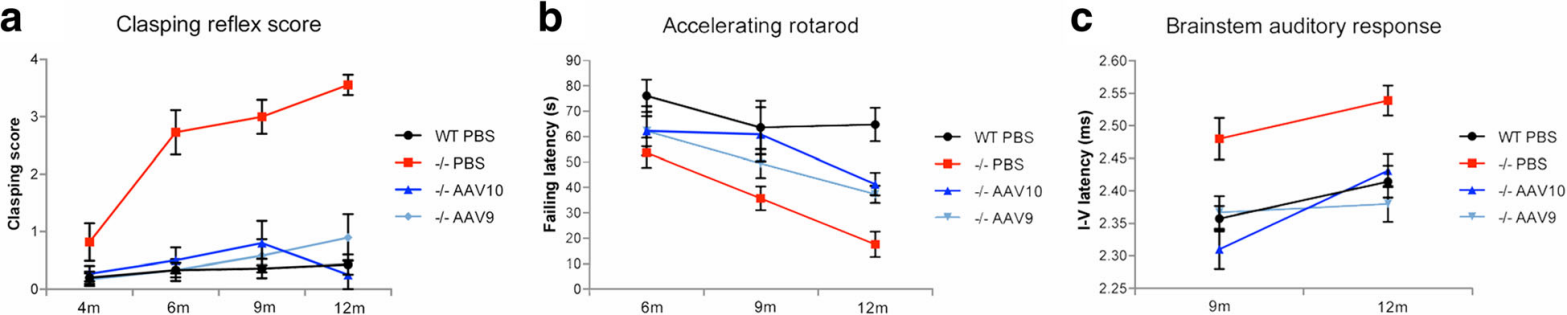
Intrathecal gene therapy provides long-term neurologic correction. Pompe mice (−/−) were injected at 1 month in the *cisterna magna* with 10^11^ vg of AAVrh10-CAG-hGAA (*n* = 12) or AAV9-CAG-hGAA (*n* = 12) or PBS (*n* = 11) and their neurologic function tests results were compared to those of wild-type (WT) mice of the same genetic background (b6;129) injected with PBS (*n* = 15). **a** Hindleg clasping reflex score from 0 (normal hindleg placement) to 4 (permanent abnormal retraction of both hindlegs). Note the rapid and progressive neurological deficit observed in mock-treated Pompe mice (−/−) PBS only (Linear mixed effects, time effect: *P* < 0.0001). **b** Coordination evaluation by accelerating rotarod test (4 to 40 rotations per minute in 3 min). **c** The latency between the first and the fifth peak of the brainstem auditory response representing a fully restored nerve conduction velocity within the auditory brainstem in treated animals (Linear mixed effects, group effect: *P* < 0,0001)

**Fig. 3:**
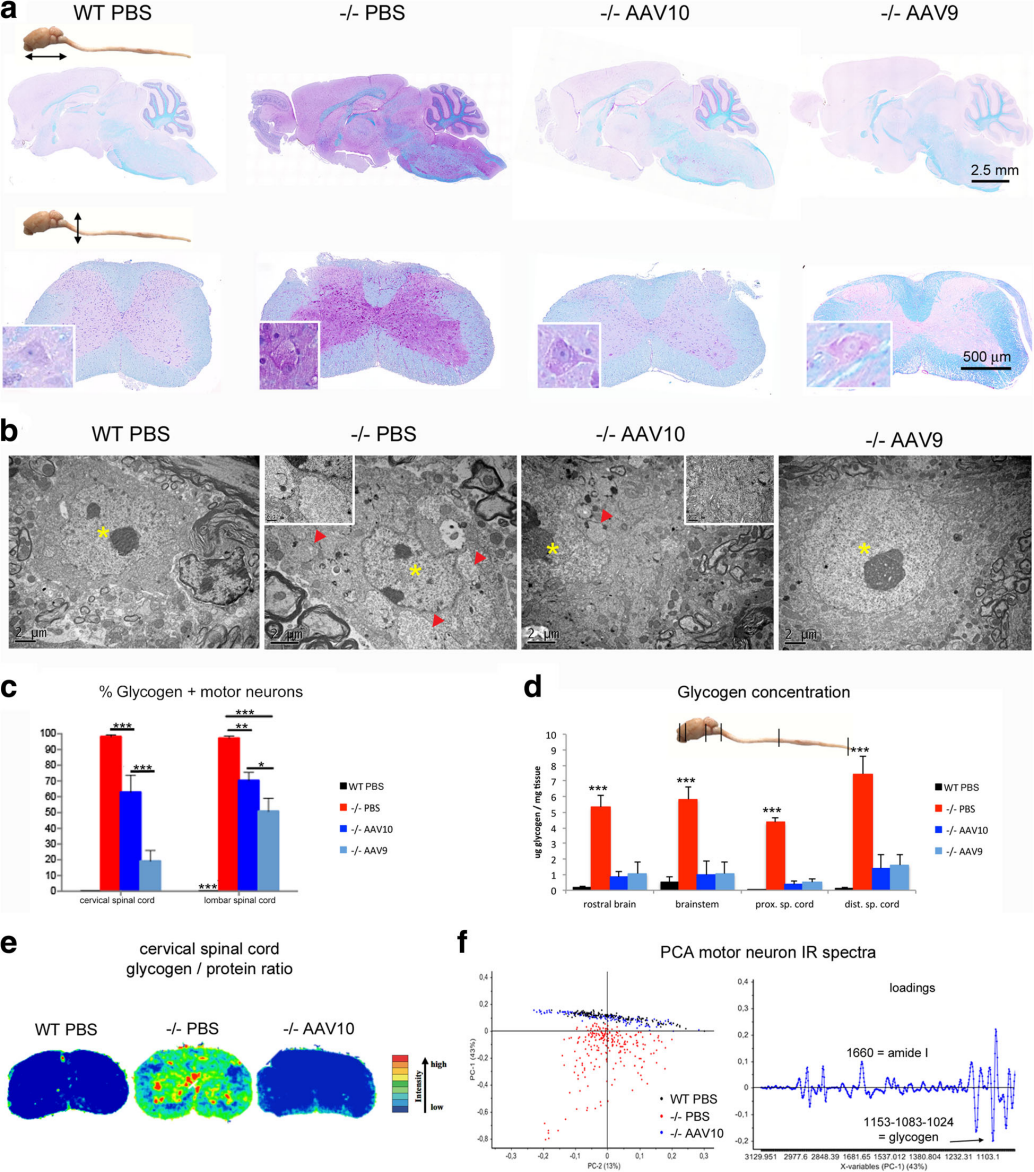
Central nervous system is normalized at 12 months. Treatment groups were as described in Fig. 1. **a** Representative sections of brain and cervical spinal cord, paraffin embedding, PAS-luxol fast blue stain. The glycogen storage appears purple on a blue background, insets show motor neuron of spinal cord ventral horn. **b** Representative ultrastructure of cervical spinal cord motor neurons, epon embedding, uranyl acetate contrast. The nuclei are indicated with yellow asterisks and the glycogenosomes with red arrowheads. **c** Quantification of the PAS positive *ie* glycogen-filled motor neurons in PAS stained paraffin-embedded sections from the cervical and lumbar spinal cord (one-way ANOVA with Newman-Keuls post hoc test; *n* = 4 animals in each group, 6 sections per animal: **P < 0.01; ***P < 0.001). **d** Glycogen concentration measurement in CNS tissue extracts obtained from samples that were snap-frozen in liquid nitrogen rapidly after sacrifice (one-way ANOVA with Newman-Keuls post hoc test; *n* = 4 to 5 per group: **P < 0.01; ***P < 0.001) ***P < 0.001). MNs were considered glycogen positives when the PAS staining intensity MN analysed was superior to PAS staining intensity of wt MNs. **(e)** Infrared chemical mapping performed on dewaxed formalin fixed paraffin embedded spinal cord sections. Chemical imaging was performed after the infrared chemical mapping showing the relative concentration of glycogen. Concentrations were determined by the area under the curve for the glycogen pic centered at 1080 cm-1 normalized versus the protein pic area centered at 1654 cm-1. **f** Principal component analysis (PCA) of the IR spectra collected by mapping motor neurons in FTIR microspectroscopy using a synchrotron light source and the corresponding loading plot (*n* = 156, 239, and 164 spectra for WT, −/− PBS, and −/− AAV10 respectively)

We then assessed the efficacy of a single intrathecal injection of AAVrh10-CAG-hGAA (*n* = 12) or AAV9-CAG-hGAA (*n* = 12) over the long term (11 months post-injection; Fig. 1). The hindleg clasping reflex scoring that assesses the proper inhibitory function of the CNS did not show any significant functional anomaly in treated mice in contrast to mock-treated mice that developed a rapid and progressive neurological deficit (Fig. 2a). The motor coordination of treated mice, assessed by accelerating rotating rod test, was improved when compared to the mock-treated (Fig. 2b) and the nerve conduction within the brainstem was fully normalized as showed by the measurements of the brainstem auditory response (Fig. 2c), demonstrating an efficient prevention of neurological manifestations onset associated with glycogen storage. Histological analyses confirmed a marked reduction of glycogen storage in the brain, the cerebellum, the brainstem, and the spinal cord of treated mice with AAVrh10 whereas serotype 9 led to a complete clearing of the storage (Fig. 3a). Consistent with the evolution towards neurological deficit, untreated mice at 12 months displayed strong glycogen storage in CNS. The motor neurons (MN), which were characterized by enlargement of the cell body and replacement of the intracytoplasmic organelles by a myriad of glycogenosomes in the mock-treated Pompe mice on electron microscopy, demonstrated a normal cell organization in AAV9-treated mice, while AAVrh10-treated mice showed a partial re-organization (Fig. 3b). Residual storage in motor neurons was quantified in the cervical and the lumbar spinal cord from AAVrh10 and AAV9-treated mice (4 animals per group and three coronal sections per location and per animal). Proportion of motor neuron reactive to PAS, with intracellular accumulation of glycogen, was significantly lower in AAV9-treated (respectively 19 ± 7% and 51 ± 8% in cervical and lumbar spinal cord) than in AAVrh10-treated (respectively 63 ± 11% and 70 ± 5% in cervical and lumbar spinal cord) Pompe mice (Fig. 3c). The disappearance of 80 to 90% of stored glycogen in the treated animals when compared to the mock-treated littermates was demonstrated in the CNS by glycogen concentration measurements (Fig. 3d and Table 1).

**Table 1:** GAA activity restoration and glycogen storage correction in the CNS 12 months after intrathecal AAV-CAG-hGAA therapy

Treatment	Tissue	Mean GAA activity (nmol/h/mg prot)	Mean GAA activity (% WT)	Mean glycogen correction relative to mock-treated (% reduction)
AAVrh10 *n* = 4	Rostral brain	1.8 (±0.6)	6.1 (±2.2)	84.3 (±6.5)
	Brainstem	35 (±21.9)	37.8 (±23.6)	83 (±15.4)
	Proximal spinal cord	10.4 (±1.7)	13.5 (±2.2)	91.9 (±5.3)
	Distal spinal cord	2.8 (±0.7)	5 (±1.2)	81.1 (±11.4)
AAV9 *n* = 4	Rostral brain	2.4 (±1.1)	8.3 (±3.9)	82.5 (±10.8)
	Brainstem	31.6 (±20.8)	34 (±22.4)	81.6 (±12.1)
	Proximal spinal cord	7.6 (±1.7)	9.9 (±2.3)	88.9 (±6.1)
	Distal spinal cord	5.1 (±0.3)	9 (±0.5)	78.3 (±8.5)

Biochemical mapping of the cervical spinal cord assessed by infrared microspectroscopy showed reduction of the glycogen in both grey and white matter from treated animals (Fig. 3e). In grey matter, the subcellular content from motor neurons was assessed by high-resolution infrared microspectroscopy using a synchrotron light source (Additional file 1: Figure S3). Principal component analysis (PCA) of spectral data from the motor neurons maps showed that both treated and WT animals were located in the same cluster characterized by the typical infrared biochemical signature of grey matter (Fig. 3f). On the contrary and consistent with our other data showing neurological deficit, the spectra of mock-treated Pompe mice were characterized by an elevation of the bands assigned to the carbohydrates of glycogen (infrared absorption bands 1152, 1080, 1025 cm^−1^). Band assignment in the infrared spectra was done according to the literature [21, 29, 51].

**Fig. 4:**
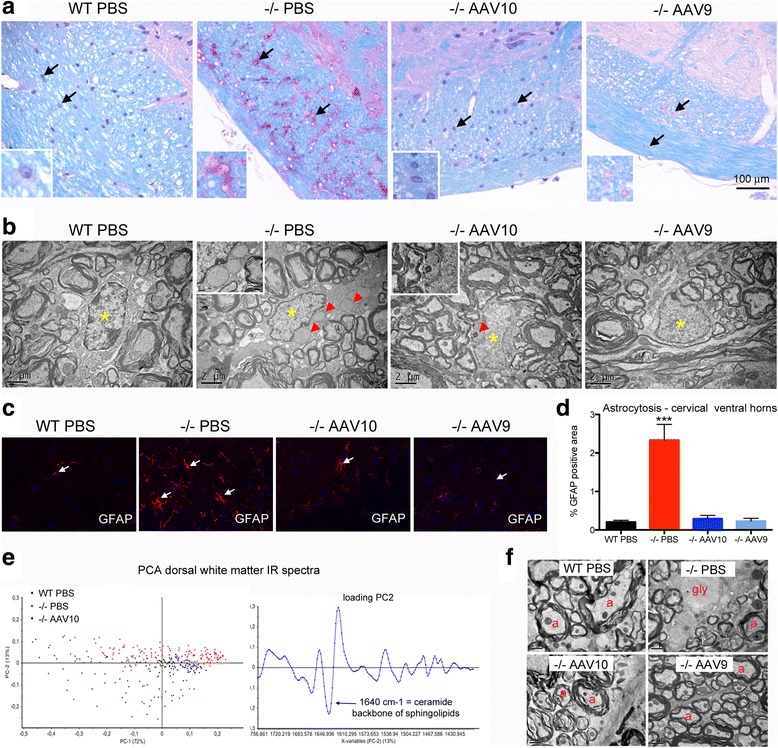
Glial cells and myelin are corrected at 12 months. Treatment groups were as described in Fig. 1. **a** Representative sections of brainstem peripheral white matter (spinal trigeminal tract), paraffin embedding, PAS-luxol fast blue stain. Arrows point to glial cells, astrocytes and oligodendrocytes. Insets show glial cells, presumably astrocytes, at a higher magnification **(b)** Representative ultrastructure of cervical spinal cord white matter oligodendrocytes, epon embedding, uranyl acetate contrast. The nuclei are indicated with yellow asterisks and the glycogenosomes with red arrowheads. **c** Representative immunofluorescence labeling of astrocytes using antibodies that recognize GFAP (glial fibrillary acidic protein) in cervical spinal cord frozen section (ventral horns). **d** Qualification of astrocytosis in cervical spinal cord ventral horns (one-way ANOVA with Newman-Keuls post hoc test; *n* = 4 per group: ***P < 0.001) **(e)** Principal component analysis (PCA) of the IR spectra collected by mapping spinal cord dorsal white matter in FTIR microspectroscopy using a synchrotron light source (*n* = 99, 149, and 94 spectra for WT, −/− PBS, and −/− AAV10 respectively) and corresponding loading plot. **f** Representative ultrastructure of cervical spinal cord dorsal white matter, epon embedding, uranyl acetate contrast. Myelinated axons (a), glycogen (gly) is seen in some demyelinated enlarged axons causing rarefaction of normal myelinated axon profiles in the mock-treated mice

Importantly, the glycogen storage correction was also observed at 12 months in the glial cells (astrocytes and oligodendrocytes) with both serotypes (Fig. 4a, b) showing that intrathecal AAV-CAG-hGAA therapy can clear both neuronal and glial storage. Reactive astrocytosis was corrected by the treatment in both groups (Fig. 4c, d). Alterations of myelin were also investigated by synchrotron FTIR microspectroscopy and by transmission electron microscopy. PCA analysis of spectral data from dorsal corticospinal white matter tracts showed white matter biochemical normalization as demonstrated by the clustering of treated and WT animals together (Fig. 4e). The normalization of the band assigned to the ceramide backbone of sphingolipids particularly abundant in myelin sheats, which is decreased in Pompe mice (infrared absorption band at 1640 cm^−1^), suggests that the treatment normalized the myelin composition. Ultrastructural analyses confirmed a preserved myelin organization in treated mice at 12 months. Animals displayed regularly arranged normal-sized myelinated axons, and no glycogen storage was seen in the white matter tracts whereas in mock-treated mice, the dorsal funiculi displayed a rarefaction of myelinated axon seen in-between lakes of glycogen (Fig. 4f).

We also investigated the glycogen storage correction in sensory neurons, especially in cervical and lumbar dorsal root ganglia. Pompe mice display almost all ganglion cells with a soma full of vacuoles. Only Pompe mice treated with AAV9 show a reduction of these vacuoles in ganglion cells of the cervical and lumbar dorsal root ganglia (Additional file 1: Figure S4).

All together, these data indicate that glycogen storage is reduced in the whole CNS 11 months after a single intrathecal GAA gene transfer in adult mice. Neuronal and glial storage, reactive astrocytosis, and demyelination are all prevented by intrathecal AAV-CAG-hGAA therapy, with better efficiency of the serotype 9 compared to the rh10.

### Intrathecal AAV-hGAA restores the CNS GAA levels over the long term

The GAA protein was detected within neurons in the whole brain from the olfactory bulbs rostrally to the *medulla oblongata* caudally by immunofluorescence (Additional file 1: Figure S5a). In the spinal cord, neurons from the dorsal root ganglia, from the dorsal horns, interneurons and motor neurons expressed GAA. Some astrocytes, oligodendrocytes, and microglial cells were also labelled with the anti-GAA antibody throughout the CNS (Additional file 1: Figure S5b). The measurement of GAA activity in the CNS revealed partial restoration in all parts that were assayed (Table 1, Additional file 1: Table S2 and Figure S6). The mean percentage of GAA activity relative to the wild type was around 8% in the rostral brain, 35% in the brainstem, and 8% in the lumbar spinal cord at 12 months. There was marked inter-individual variations especially in the brainstem (from 4 to 105%, Additional file 1: Table S2) and no significant difference was seen between the 2 serotypes of AAV that were administered. Vector genome (vg) copies quantified by qPCR were detectable in the brainstem of treated animals with inter-individuals variations: 0.1 to 0.4 vg per diploid genome (vg/dg) with AAV9 and 0.01 to 11.31 vg/dg with AAVrh10. No copy was detected at distance from the injection site in the lumbar spinal cord (sensitivity of the assay 0.002 vg/dg) whereas the GAA protein was detected in those distal spinal segments. The protein detected in the lumbar spinal cord [3.64 ± 1.80 ng/mg (*n* = 4) in the AAVrh10 group and 6.65 ± 1.76 ng/mg (*n* = 4) in the AAV9 group was thus probably secreted from the proximal spinal cord.

### Long-term neurological correction leads to an improvement of the global muscle strength

**Fig. 5:**
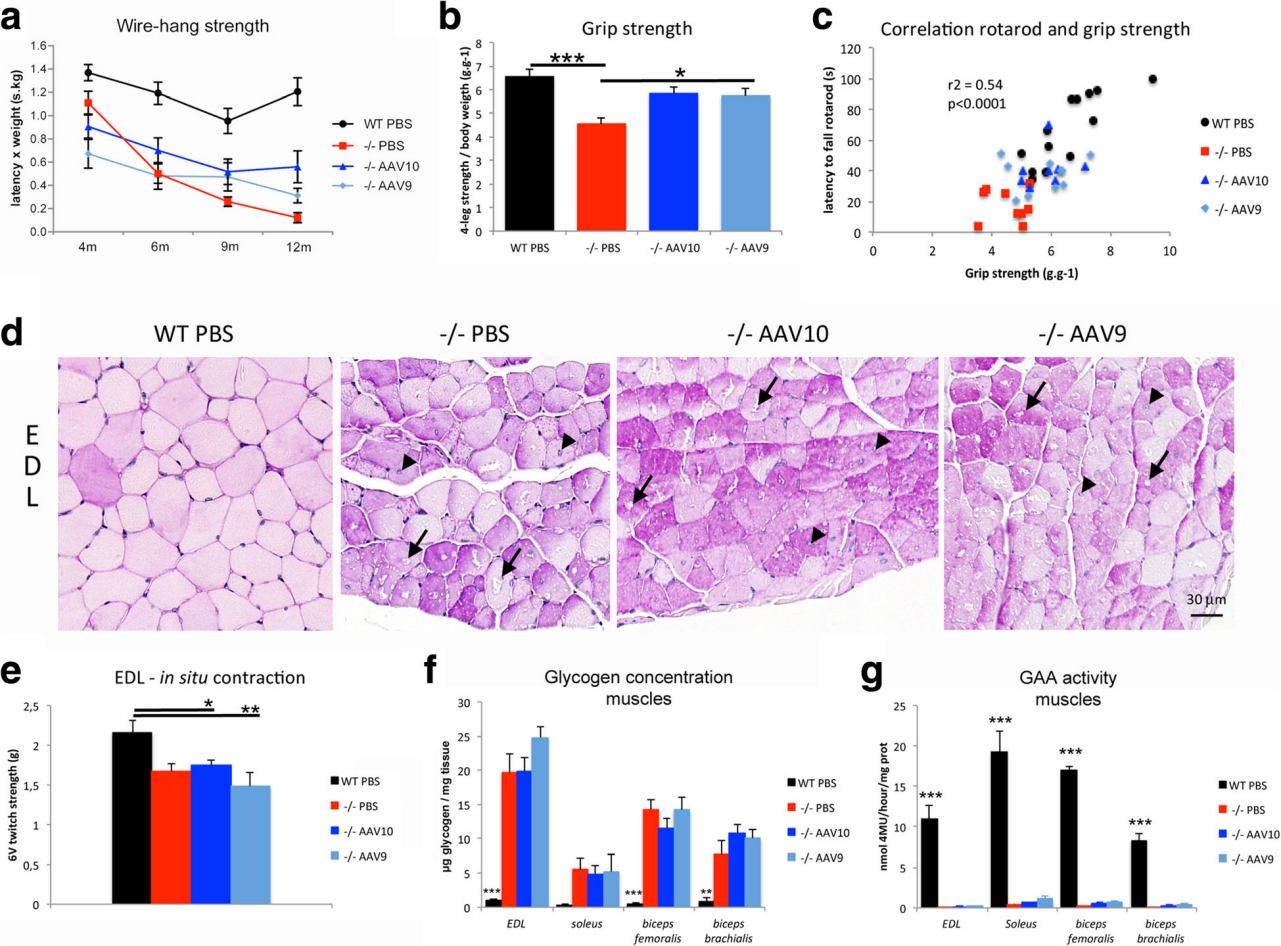
Strength is partially improved in the absence of muscular pathology correction. Treatment groups were as described in Fig. 1. **a** Wire-hang strength measures the ability of the mice to hold their own weight when suspended to a wire screen. Mean latency to fall is multiplied by the weight of the mice (Linear mixed effects, time effect: *P* < 0.0001; *n* = 8 to 13 per group). **b** Four-legs grip strength measurement using a grip meter and a force transducer (one-way ANOVA with Newman-Keuls post hoc test; *n* = 8 to 13 per group: *P < 0.05, ***P < 0.001) **(c)** Correlation between the accelerating rotarod latency and the grip strength. **d** Representative cross sections of *extensor digitorum longus* muscle (EDL), paraffin embedding, PAS stain. The glycogen storage appears purple. Arrows point to vacuoles in the cytoplasm of the fibers and arrowheads point to central nuclei. **e** In situ contraction study, maximal twitch tension developed by the EDL after 6 V stimulation (one-way ANOVA with Newman-Keuls post hoc test; *n* = 8 to 12 per group: *P < 0.05, **P < 0.01). **f** Glycogen concentration measurement in muscle tissue extracts obtained from samples that were snap-frozen in liquid nitrogen rapidly after sacrifice (one-way ANOVA with Newman-Keuls post hoc test; *n* = 4 to 5 per group: **P < 0.01, ***P < 0.001) **(g)** GAA enzymatic assay in the same extracts using 4-methylumbelliferyl-α-D-glucopyranoside as substrate (one-way ANOVA with Newman-Keuls post hoc test; *n* = 4 to 5 per group: ***P < 0.001)

We hypothesized that in case of successful CNS correction, a positive effect upon the muscle strength of the mice would be measurable. Mixed neuromuscular tests (wire-hang strength and grip strength), muscular primary pathology, and in situ muscular twitch tension recordings were compared in order to discriminate the respective impact of the CNS and of the muscles over the global strength. The global strength of the mice, assessed by the wire-hang and the grip test measurements, was greatly improved in both groups of treated mice showing strength values similar than the WT (Fig. 5a, b). Interestingly, we show a link between the motor coordination improvement (due to the CNS pathology rescue) and the strength improvement as demonstrated by a positive correlation between the rotarod time latencies and the grip strength developed by the mice (r^2^ = 0.54 *p* < 0.0001, Fig. 5c). Muscle fibers of both mock-treated and AAV-treated mice were vacuolated (Fig. 5d) and atrophied (Additional file 1: Figure S7) suggesting that the improvement in global strength was not due to cross-correction of the muscular primary pathology by blood circulating GAA. Accordingly, the in situ contraction study showed that the maximal twitch tension developed by the *extensor digitorum longus* muscle in Pompe mice was significantly decreased and was not restored by the treatment (Fig. 5e). In addition, the GAA enzymatic activity was not restored in the muscles of treated mice and the glycogen storage was severe confirming the absence of muscular pathology correction (Fig. 5f, g).

Gathering the neuromuscular and muscular data together, the absence of muscular primary pathology rescue suggests that the strength improvement is solely related to the correction of the CNS by intrathecal gene therapy.

### Intrathecal AAV9-hGAA improves the hypertrophic cardiomyopathy

**Fig. 6:**
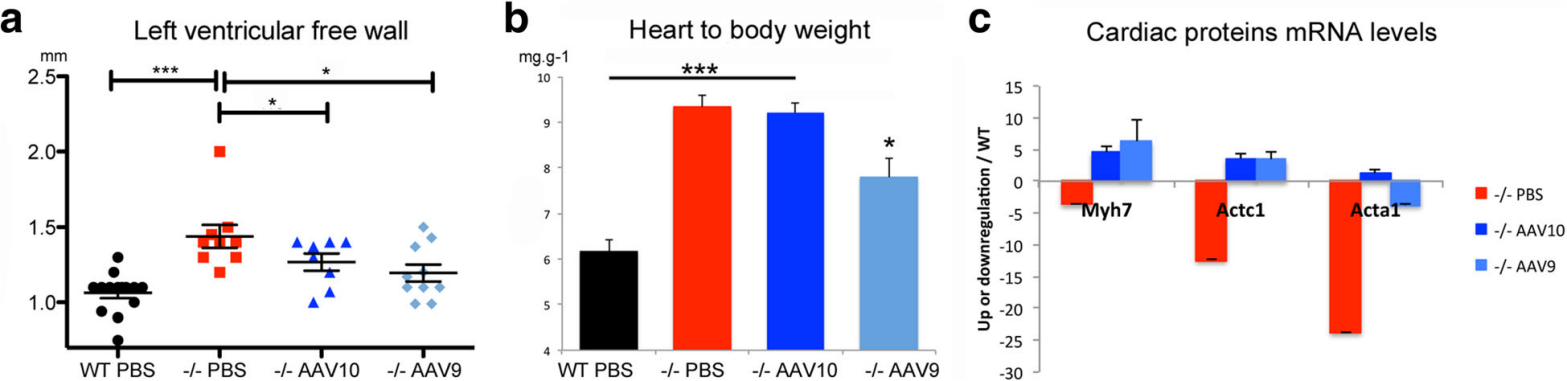
Intrathecal administration of AAV9-CAG-hGAA improves cardiac hypertrophy and both serotypes increase the gene expression of cardiac structural proteins. **a** Left ventricular free wall thickness measurement at the end of diastole by echocardiography M-mode (one-way ANOVA with Newman-Keuls post hoc test; *n* = 8 to 10 per group: **P < 0.01, ***P < 0.001). **b** Heart to body weight ratio at euthanasia (one-way ANOVA with Newman-Keuls post hoc test; *n* = 8 to 13 per group: *P < 0.05, ***P < 0.001). **c** Cardiac structural proteins transcript quantification, mRNA up- or down-regulation relative to WT levels (*n* = 4 per group). Myosin beta heavy chain 7 of cardiac muscle (myh7), actin alpha cardiac muscle 1 (actc1), and actin alpha 1 (acta1). (one-way ANOVA with Newman-Keuls post hoc test; *n* = 8 to 13 per group: *P < 0.05, **P < 0.01)

Improvement of the hypertrophic cardiomyopathy was obtained in AAV9 treated animals as shown by a significant reduction of the thickness of the left ventricular wall (Fig. 6a) and by improvement of the heart to body weight ratio (Fig. 6b). Myosin beta heavy chain 7 (myh7), actin alpha cardiac muscle 1 (actc1) and actin alpha 1 (acta1) genes that are known to be involved in the hypertrophic remodeling of the cardiac muscle, were downregulated in the mock-treated Pompe mice despite clinical cardiac hypertrophy, showing that the pathogenesis of Pompe’s cardiomyopathy is related to the storage rather than to a sarcomeropathy. Expression of these three cardiac genes seemed to be increased in treated cardiomyofibers (Fig. 6c).

**Fig. 7:**
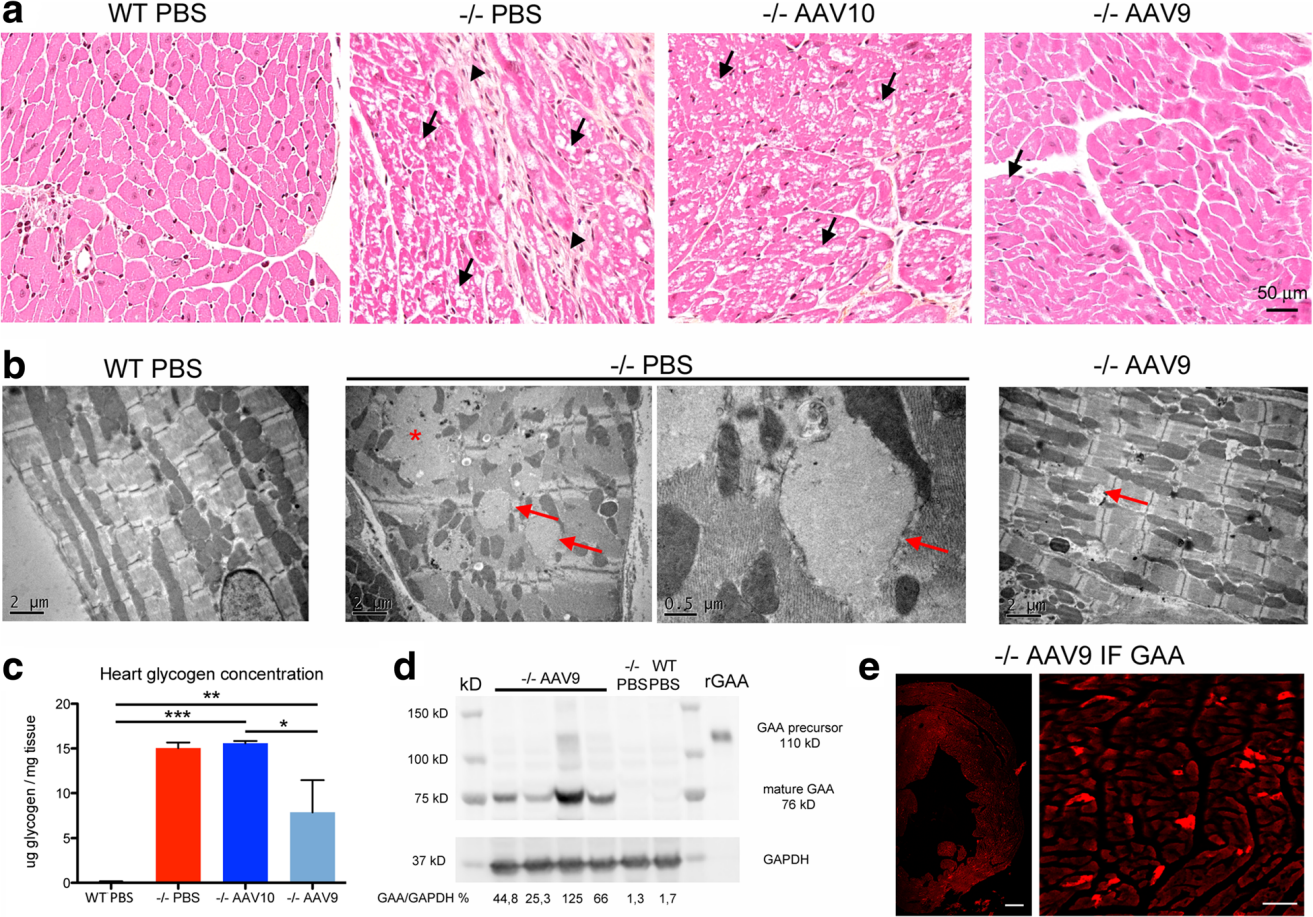
Cardiac muscle pathology improvement is correlated to the presence of GAA. **a** Representative cross sections of the left ventricular free wall, paraffin embedding, hemalun-eosin-saffron stain. The collagen appears orange on this trichrome stain. Arrows point to vacuoles in the cytoplasm of the fibers and arrowheads point to fibrosis. The cardiac muscle pathology is improved with AAV9 only. **b** Representative ultrastructure of the left ventricular free wall, epon embedding, uranyl-acetate contrast. Arrows point to glycogenosomes and the asterisk shows a “glycogen lake” that occurs after rupture of enlarged lysosomes. **c** Glycogen concentration measurement in heart extracts obtained from samples that were snap-frozen in liquid nitrogen rapidly after sacrifice (one-way ANOVA with Newman-Keuls post hoc test; *n* = 4 to 5 per group: **P < 0.01, ***P < 0.001) **(d)** Western-blot analysis of heart tissue extracts with a rat polyclonal anti-rGAA antibody. Glyceraldehyde-3-phosphate dehydrogenase (GAPDH) immunodetection is used as a protein loading control. **e** Representative immunofluorescence labeling of GAA in AAV9 treated mice using a rat polyclonal anti-rGAA antibody on a frozen heart section. Scale bars = 200 μm

The severe glycogen accumulation causing vacuolation and hypertrophy of the cardiac fibers was corrected in AAV9 treated mice only (Fig. 7a). Electron microscopy analysis demonstrated in mice treated with AAV9 near complete disappearance of the glycogen storage, well arranged myofibrils, and a characteristic location of mitochondria between the myofibrils whereas mock-treated mice displayed a complete disorganization of the myofibrils and mitochondria due to the presence of glycogen in enlarged lysosomes or free in the cytoplasm after lysosomal rupture (Fig. 7b). Accordingly, the dosage of glycogen showed reduction of storage in AAV9 treated mice only (Fig. 7c, Additional file 1: Table S2).

Mature 76kD GAA was readily blotted from the hearts of AAV9 treated mice, showing that GAA was correctly addressed and processed within cardiac fibers (Fig. 7d) whereas only weak or absent bands were observed in AAVrh10 treated mice (not shown). GAA was also observed in the hearts by immunofluorescence (Fig. 7e). Titration of GAA by ELISA confirmed the results of GAA activity, i.e. better enzymatic restoration in the hearts of AAV9 treated mice: 25.81 ± 8.17 ng/mg proteins was measured in the AAV9 group and 6.97 ± 1.69 ng/mg proteins of GAA in the AAVrh10 group (*p* < 0.05 *n* = 4 per group).

Vector genome was not recovered from hearts by qPCR (<0.002 vg per diploid genome) except for one in the AAV9 group with 0.004 vg/dg, suggesting that cardiac GAA was uptaked from the circulation. The liver was transduced in all tested animals. The transduction with AAV9 was twice higher than with AAVrh10 (4.61 ± 0.80 vg/dg in the AAV9 group and 2.36 ± 0.50 vg/dg in the AAVrh10 group; *p* < 0.05 *n* = 4 per group), which could explain the difference between serotypes for the GAA secretion and thus the cardiac correction.

At 12 months, anti GAA antibodies were detected by indirect ELISA in sera dilution from 1/100 to 1/10,000 in AAV9 treated animals and from 1/100 to 1/100,000 in AAVrh10 treated animals (Additional file 1: Figure S8). Comparison of mean titers between serotypes revealed no significant difference (*p* = 0.1; *n* = 8 AAVrh10 and *n* = 9 AAV9), suggesting that immunization levels did not explain the lack of cardiac cross-correction in the AAVrh10 group. We hypothesized that the composition of the capsid could lead to specific behaviour of the viral particles regarding the redistribution from the cerebrospinal fluid (CSF) into the systemic circulation and/or the persistence of the particles in the blood. Viral particles were 6 times more abundant in the blood 1 h after the injection of AAV9 compared to AAVrh10 (mean results 2.77 × 10^7^ vg AAVrh10/5 μl serum and 1.59 × 10^8^ vg AAV9/5 μl serum, *n* = 4 per serotype; *p* = 0.06). Particles of AAV9 were moreover present for up to 7 days while AAVrh10 was not measurable with our assay at 7 days post-injection (Additional file 1: Figure S9; sensitivity of the assay 2 × 10^3^ vg/5 μl serum). If we express those results as percentage of the total administered dose (considering 0.5 ml of serum per adult mouse), 2.0 ± 0.7% of total AAVrh10 particles and 11.8 ± 4.0% of total AAV9 particles were in the systemic circulation 1 h after the intrathecal administration. Those results suggest that AAV9 was redistributed in the systemic circulation and eliminated from the organism at a slower rate. Therefore, the difference between AAV9 and AAVrh10 regarding peripheral cross-correction could be related to the circulation of AAV9 particles in the blood for a longer period of time, allowing better hepatic transduction.

## Discussion

We demonstrate that an intrathecal gene replacement therapy can lead to a long-term global central nervous system correction, prevention of the neurological deficits, improvement of the neuromuscular function, and alleviation of the hypertrophic cardiomyopathy in a Pompe disease model. Delivery of the therapeutic recombinant AAV vector into the cerebrospinal fluid at the age of 1 month, when lysosomal pathology is already advanced in the CNS, allowed full neurological, partial neuromuscular and cardiac correction for 1 year. Importantly no treatment related adverse reaction or toxicity has been observed.

Classic infantile Pompe disease is characterized by the accumulation of glycogen-loaded lysosomes causing cellular hypertrophy, the disorganization and rarefaction of organelles, and cell dysfunction especially in the heart, skeletal muscles, and the CNS [30]. Within the CNS, motor neurons of the brainstem and spinal cord, sensory neurons of the brainstem and dorsal root ganglia, and also glial cells, are the most severely affected [39, 62]. Such motor and sensory neuron impairment is now increasingly recognized as the cause of a progressive neurologic phenotype in patients under enzyme replacement therapy [5, 48, 67]. Dysfunction of the motor neurons in the brainstem causes facial and bulbar muscle weakness with speech and swallowing disorders as a clinical consequence [67]; anterior horns neurons involvement can lead to motor polyneuropathy and motor unit dysfunction [5, 15]. Moreover GAA deficiency in the central nervous system contributes to respiratory deficiency [26, 65]. These recent observations highlight the necessity to propose a global muscular and neurological directed therapy to Pompe disease patients. Our strategy efficiently reverses the glycogen accumulation throughout the brainstem and spinal cord, which are the most severely affected regions in the CNS of infantile Pompe disease patients [39]. The accumulation of glycogenosomes is cleared and the organelles are restored in all affected cells i.e. the motor neurons, the sensory neurons, and the glial cells. With a translational goal in mind, our results demonstrate that low levels of GAA restoration in the CNS, around 10% of the physiological levels, are sufficient to correct the lysosomal pathology in neurons and glial cells. This is in accordance with the absence of neuronal storage in the patients with juvenile and adult onset of the disease [31] and with what is known for other neurological LSD [13]. This should allow adjusting the minimal posology in order to ensure the safety of CNS directed GAA gene transfer in the future clinical trials. The feasibility of AAV-mediated gene transfer via the CSF in large animal models and non-human primates has been largely demonstrated by we [3, 4], and others [19, 25, 27, 28, 41, 42, 49, 50].

Correction of the CNS pathology in our study leads to clinical neurological normalization measured by the disappearance of the clasping reflex, the restoration of nerve conduction within the auditory brainstem, the improvement of the coordination and the global neuromuscular function. Neither AAV transduction, nor GAA activity in muscles has been reported in our study after AAV9 or 10 treatments, highlighting the neurogenic muscle weakness and the pathology of the neuromuscular junction also reported by others [15, 24, 63]. However, several studies have demonstrated a reduction of glycogen content in striated muscles and preservation of muscle strength due to extensive transgenic GAA production in liver after systemic AAV or adenoviral delivery without correction of the CNS, suggesting that neural transduction is not required to improve strength [33, 58, 60, 71]. Nevertheless, we shared the hypothesis advanced by Byrne’s group notably, that therapies targeting both skeletal muscle and CNS may be needed [6] to obtain a full recovery. Interestingly, some studies have demonstrated GAA activity in brain following AAV8 systemic administration in GAA KO mice [59]. However a slight reduction, only, in glycogen storage was reported in non-immunocompetent mice [68], even with beta-2 agonists adjunction, which could favor the transfer through the blood brain barrier [38]. For systemic administration, the development of a humoral immune response remains an issue, hampering maintenance of the metabolic correction [17].

The current approved treatment, ERT, efficiently restores cardiac function but does not allow neurological correction due to the blood-brain-barrier [45]. Infantile Pompe disease patients under ERT thus demonstrate a unique phenotype characterized by a persistent muscular weakness in specific group of muscles that are usually not commonly involved in late onset Pompe disease: facial and bulbar muscles, neck flexor, dorsiflexor, and hip extensor muscles [11]. This selective weakness might be related to the storage in selective groups of motor neurons. In the murine model, we observed that the storage in the motor neurons of the brainstem is earlier and more pronounced than in anterior horn motor neurons. Moreover, experimental data obtained in the murine model recently demonstrated that the storage of phrenic motor neurons and hypoglossal motor neurons is involved in the respiratory muscles and tongue weakness respectively [18, 37, 44, 65]. Indeed the correction of phrenic motoneurons can increase ventilation in Pompe mice [23, 44]. Recently the first clinical trial of diaphragmatic gene therapy has successfully treated respiratory neural dysfunction in infantile Pompe patients [8, 55, 56]. The strength improvement of intrathecally AAV-hGAA treated mice in our study, despite uncorrected muscular pathology, adds new arguments in favor of the CNS implication in the physiopathology of infantile Pompe disease. This means that future therapies will have to address both muscular and neurologic manifestations of the disease. We propose that the intrathecal administration of the vector encoding GAA could be performed concurrently with the first ERT administrations, or shortly after, or in combination with a systemic AAV gene therapy. Our results that demonstrate a better efficiency of AAV9 for the correction of hypertrophic cardiomyopathy, and the use of AAV9 in a CNS-directed trials in human (Spinal Muscular Atrophy NCT02122952) lead us to choose this serotype for human translation. According to our study of viral particles distribution and persistence in the blood after intrathecal administration, serotype 9 has a slow kinetic of clearing from the bloodstream that allows more robust liver transduction, and consequently the secretion of more transgenic GAA into the systemic circulation. The unique persistence of AAV9 viral particles into the circulation has already been demonstrated by others [52]; it seems to be a feature of the serotype 9 solely, and to rely on the galactose receptor footprint [53]. This allowed the rescue of supraphysiological GAA activity, the normalization of cardiac ultrastructure, and the reduction of cardiac glycogen storage in the heart of AAV9-treated mice

Prior to the assessment of a combined ERT and intrathecal gene therapy in infantile patients, we are currently designing a non-human primate study that will explore both CNS and peripheral transduction, and the potential consequences of GAA overexpression. If our results safely translate to the non-human primate, intrathecal administration of AAV9-CAG-hGAA to infantile Pompe disease patients should allow to correct the CNS and to transduce the liver that will act as a continuous source of transgenic GAA. CNS GAA restoration would offer neuromuscular and strength improvement while the continuous secretion of GAA from the liver should allow decreasing the dose of recombinant enzyme and/or to space out the injections.

## Conclusions

To conclude, the preclinical data presented here demonstrate that intrathecal gene therapy is an attractive and promising strategy for the management of the increasingly acknowledged neurological impairment in infantile Pompe disease patients.

## Additional file

Additional file 1: Supplementary datas. (DOCX 44817 kb)
