# Supplementary material for: Long-term neurologic and cardiac correction by intrathecal gene therapy in Pompe disease
Source: Acta Neuropathol Commun. 2017 Sep 6;5:66. doi: 10.1186/s40478-017-0464-2 (PMC5585940; doi:10.1186/s40478-017-0464-2)
Supplement: Additional file 1: — Supplementary datas. (DOCX 44817 kb) [file 40478_2017_464_MOESM1_ESM.docx]

Supplementary Materials

Long-term neurologic and cardiac correction by intrathecal gene therapy in Pompe disease

J. Hordeaux^1,2,3^, L. Dubreil^1,2^ , C. Robveille^1,2^, J. Deniaud^1,2^, Q. Pascal^1,2^, B. Dequéant^1,2^, J. Pailloux^1,2^, L. Lagalice^1,2^, M. Ledevin^1,2^, C. Babarit^1,2^, P. Costiou^2^, F. Jamme^4^, M. Fusellier^5,^, Y. Mallem^6^, C. Ciron^1,2^, C. Huchet^3,7^, C. Caillaud^8,9^, and M-A Colle^1,2^.

^1^INRA UMR U703, Animal Pathophysiology and Biotherapy for Muscle and Nervous system Diseases, Nantes, F-44307, France.

^2^LUNAM université, Oniris, Nantes-Atlantic national college of veterinary medicine, food science and engineering, CS 44706, Nantes, F-44307, France.

^3^LUNAM université, Université de Nantes, UFR Sciences et Techniques. Nantes, F-44322, France.

^4^SOLEIL French national synchrotron facility, Gif-sur-Yvette, F-91192, France.

^5^LUNAM université, Oniris, Department of Medical Imaging, Centre de Recherche et d’Investigation Préclinique, Nantes, F-44307, France.

^6^LUNAM université, Oniris, Physiopathologie Animale et Pharmacologie Fonctionnelle, Nantes, F-44307, France.

^7^INSERM UMR1087/ CNRS UMR6291, l’Institut du Thorax, Nantes, F-44322, France.

^8^INSERM UMR1151/CNRS UMR8253, Institut Necker Enfants Malades, Paris, F-75993, France.

^9^Université Paris Descartes, Paris, F-75006, France.

Corresponding author:

Marie-Anne Colle

Animal Pathophysiology and Biotherapy for Muscle and Nervous system Diseases,

UMR 703 PAnTher INRA/ONIRIS,

ONIRIS, CS 40706, F-44307 Nantes Cedex 03, FRANCE

Phone: 33-2-40687807 ; fax: 33-2-40180002

Email: marie-anne.colle@oniris-nantes.fr

Fig. S1


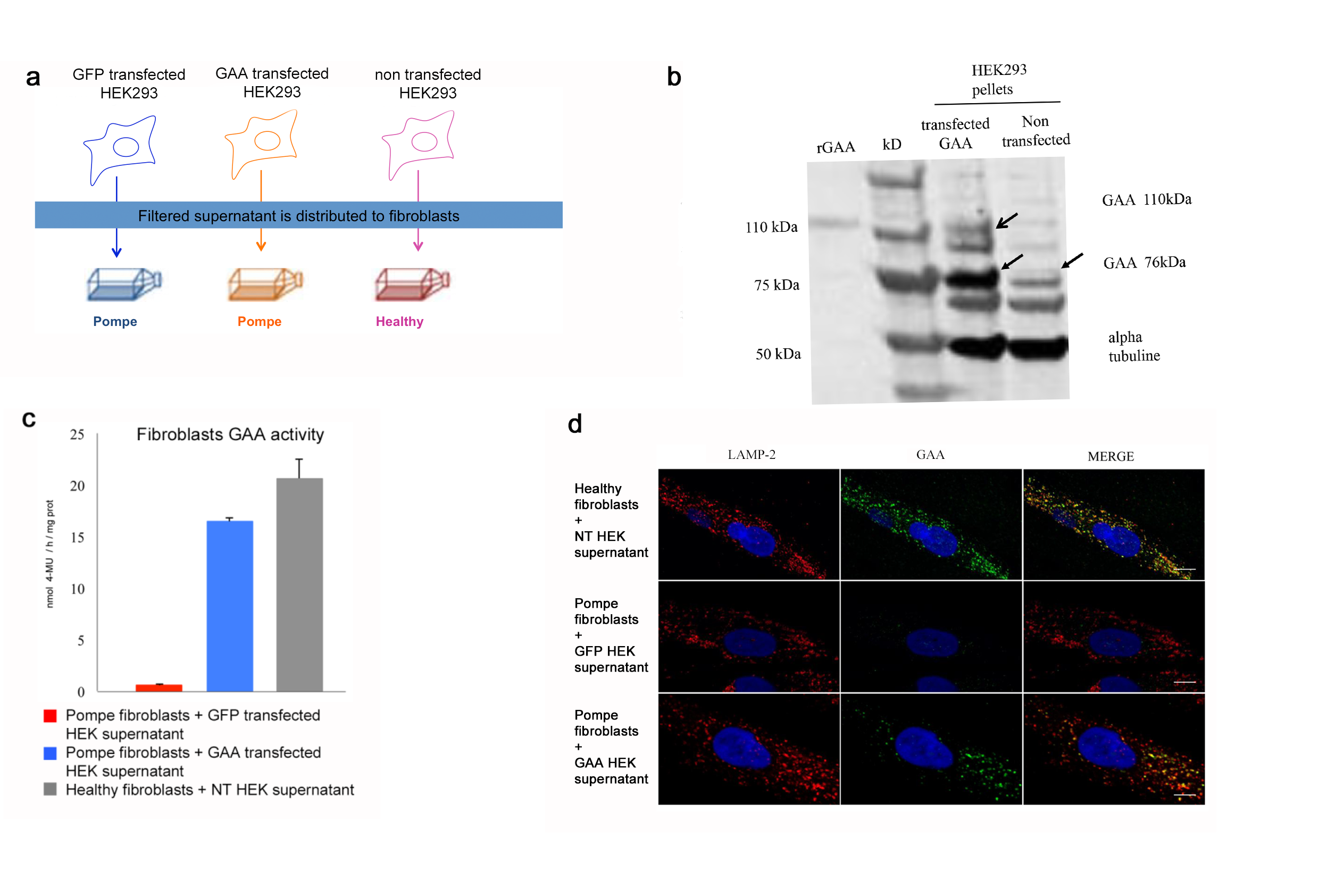


**Fig. S1** *In vitro* validation of the CAG-hGAA plasmid by transfection of HEK293 cells, and secretion-uptake assay on fibroblasts of patients

**(a)** Design of the assay. HEK293 cells were transfected with plasmids coding GFP or GAA under the control of the CAG promoter. The supernatant of HEK293 were then filtered and distributed to the fibroblasts of Pompe patients or healthy patients for 3 consecutive days. **(b)** The overexpression of GAA was verified in the HEK293 pellets by western blot analysis using a rabbit polyclonal anti-rGAA antibody. Alpha-tubuline immunodectection was used as protein loading control. GAA is overexpressed in CAG-gaa transfected HEK293. **(c)** The fibroblasts were cultured in presence of the HEK293 supernatants as showed on (a) and cell pellets were assayed for GAA enzymatic activity using 4-methylumbelliferyl-α-D-glucopyranoside as substrate (mean of 3 independent experiments). The fibroblasts of Pompe patients efficiently uptake extracellular GAA to restore 70% of the activity measured in fibroblasts of healthy patient. **(d)** Immunofluorescence detection of LAMP-2 (protein of the lysosomal membrane) and GAA in the fibroblasts. GAA is internalized by the cells and reaches the lysosomes. Scale bars = 10 μm.

**Fig. S2**

**
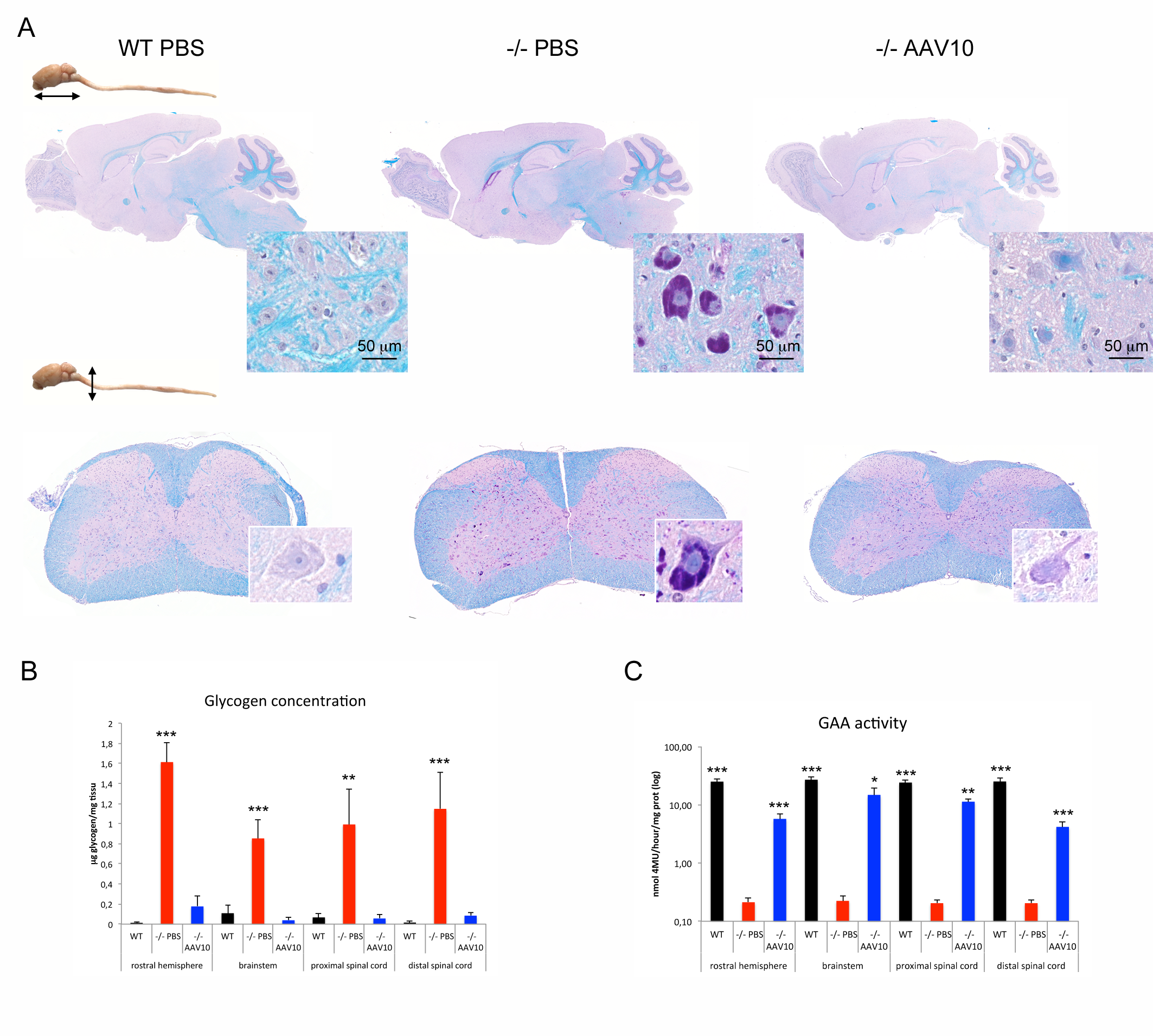
**

**Fig. S2** The CNS is corrected 3 months after intrathecal AAVrh10 administration

Pompe mice (-/-) were injected at one month in the *cisterna magna* with 10^11^ vg of AAVrh10-CAG-hGAA (n=14) or PBS (n=14) and sacrificed at 4 months of age. **(a)** Representative sagittal sections of brain (inset from brainstem) and cross-sections of cervical spinal cord (inset of a motor neuron, from the ventral horn), paraffin embedding, PAS-luxol fast blue stain. The glycogen storage appears purple on a blue background. **(b)** Glycogen concentration measurement in CNS tissue extracts obtained from samples that were snap-frozen in liquid nitrogen rapidly after sacrifice (one-way ANOVA with Newman-Keuls *post hoc* test; n=9 WT, 7 -/- PBS, and 8 -/- AAV10: **P<0.01, ***P<0.001). **(c)** GAA enzymatic assay in the same extracts using 4-methylumbelliferyl-α-D-glucopyranoside as substrate. (one-way ANOVA with Newman-Keuls *post hoc* test; n=9 WT, 7 -/- PBS, and 8 -/- AAV10: *P<0,05, **P<0.01, ***P<0.001) .

**Fig. S3**

**
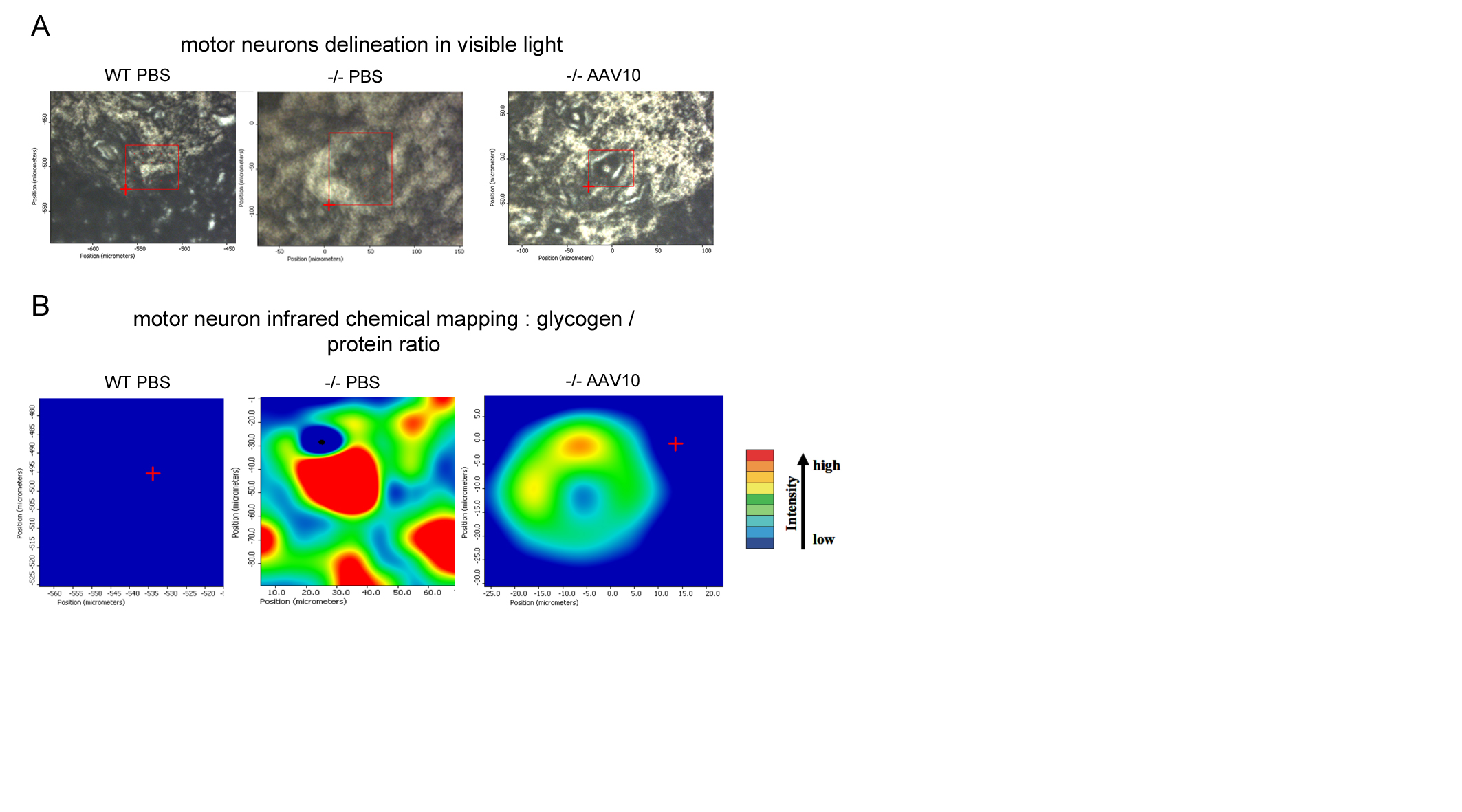
**

**Fig. S3** Chemical mapping of motor neurons by Fourier transform infrared (FTIR) miscrospectroscopy using a synchrotron light source

**(a)** The microscope was used in brightfield mode to delineate the motor neurons in the ventral horns from dewaxed formalin fixed paraffin embedded cervical spinal cord unstained sections **(b)** The chosen area was then mapped after IR illumination with a synchrotron light source (French Synchrotron Soleil facility) and collection of the absorption spectra (aperture 10 μm, step 5 μm). The glycogen content was normalized versus the proteins content by performing the ratio between glycogen pic area centered at (1080 cm-1) and protein pic area centered at 1654 cm-1. The glycogen content is lower in the treated motor neuron compared to the non-treated motor neuron.

**Fig. S4**


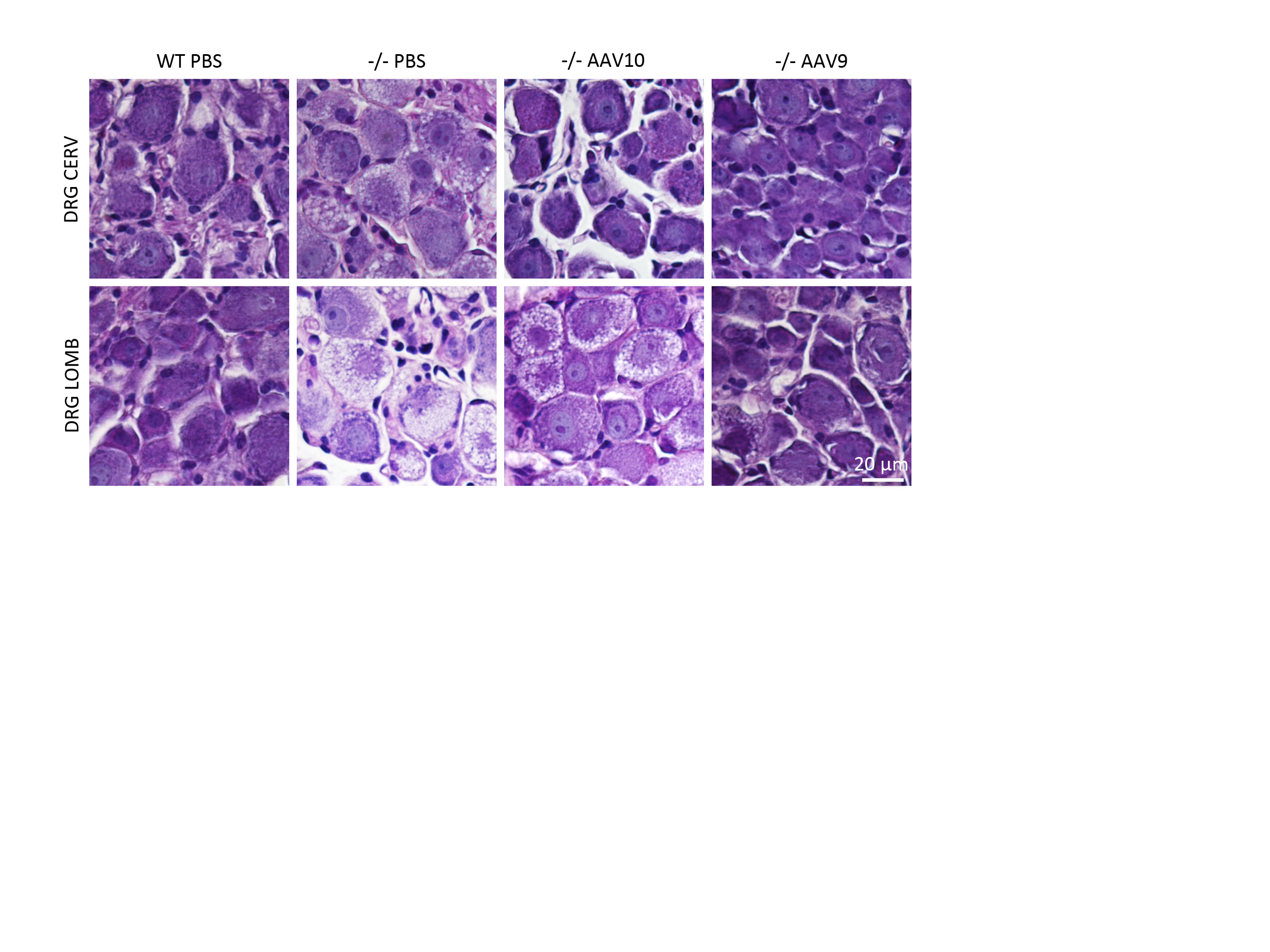


**Fig. S4** Sensory neurons from cervical and lumbar dorsal root ganglia are almost corrected at 12 months in AAV9-treated mice

Treatment groups were as described in Fig. 1. Representative sections of cervical and lumbar dorsal root ganglion, paraffin embedding, PAS-luxol fast blue stain. Both AAV9 and AAV10-treated mice show a clear reduction of vacuoles in cervical dorsal root ganglia, with a partial reduction in lumbar dorsal root ganglia in AAV9-treated mice only.

**Fig. S5**

**
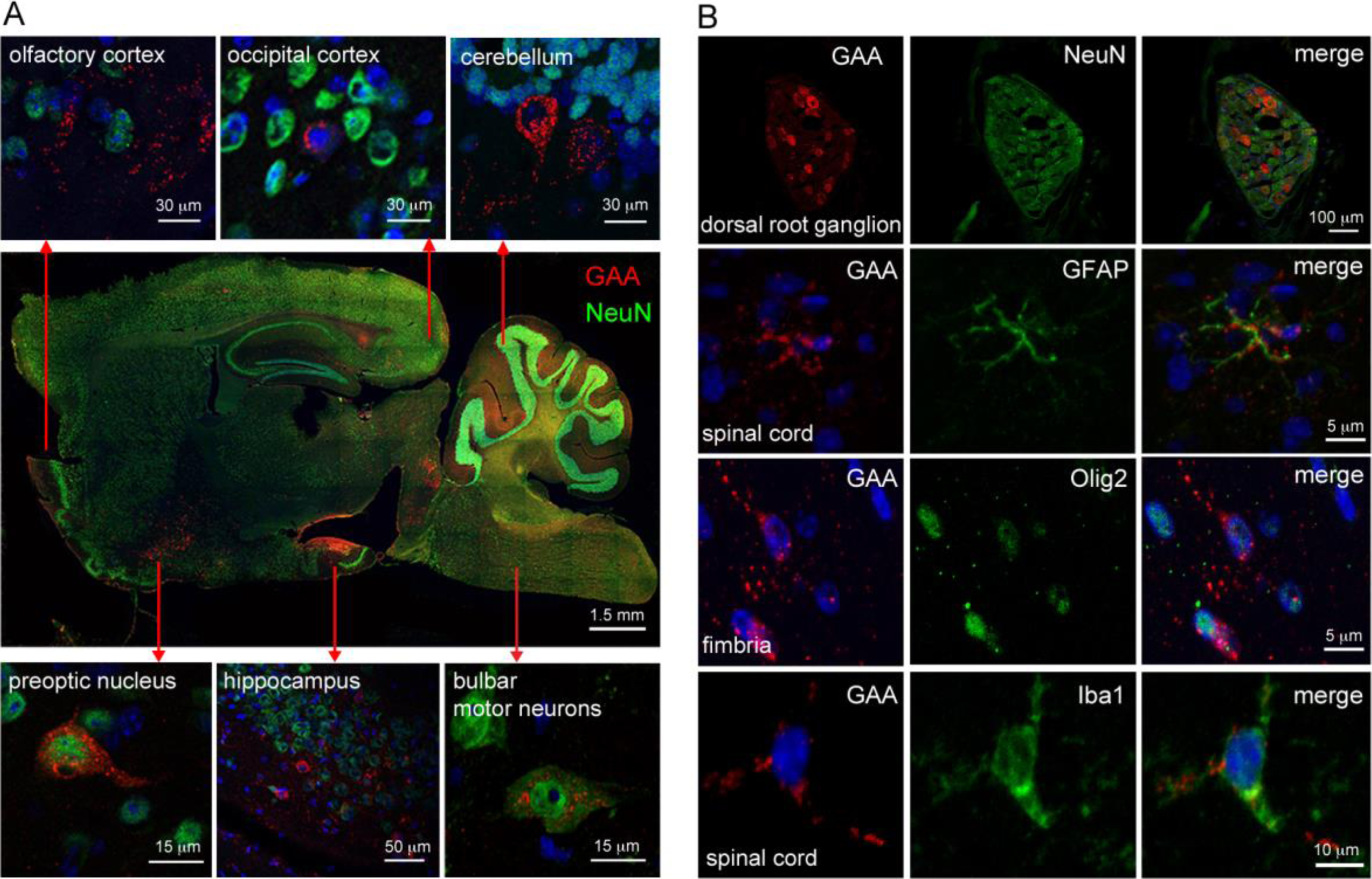
**

**Fig. S5** GAA immunofluorescence detection within the central nervous system at 12 months

Representative cryosections of an animal injected with 10^11^ vg of AAV9-CAG-gaa in the cisterna magna at one month. **(a)** Immunofluorescence study of GAA (red immunolabeling) and NeuN (neuronal marker, green immunolabeling) on brain sagittal cryosection. GAA is seen in the cytoplasm of neurons in the olfactory cortex, the occipital cortex, the cerebellum (Purkinje cells), the preoptic nucleus, the hippocampus (dentate gyrus), and bulbar motor neurons. **(b)** Immunofluorescence colocalization study of GAA (rabbit or rat polyclonal antibody, red) with NeuN (neurons, green), GFAP (astrocytes, green), Olig 2 (oligodendrocytes, green), or Iba1 (microglial cells, green). GAA is detected in some neurons, astrocytes, oligodendrocytes, and microglial cells.

**Fig. S6**

**
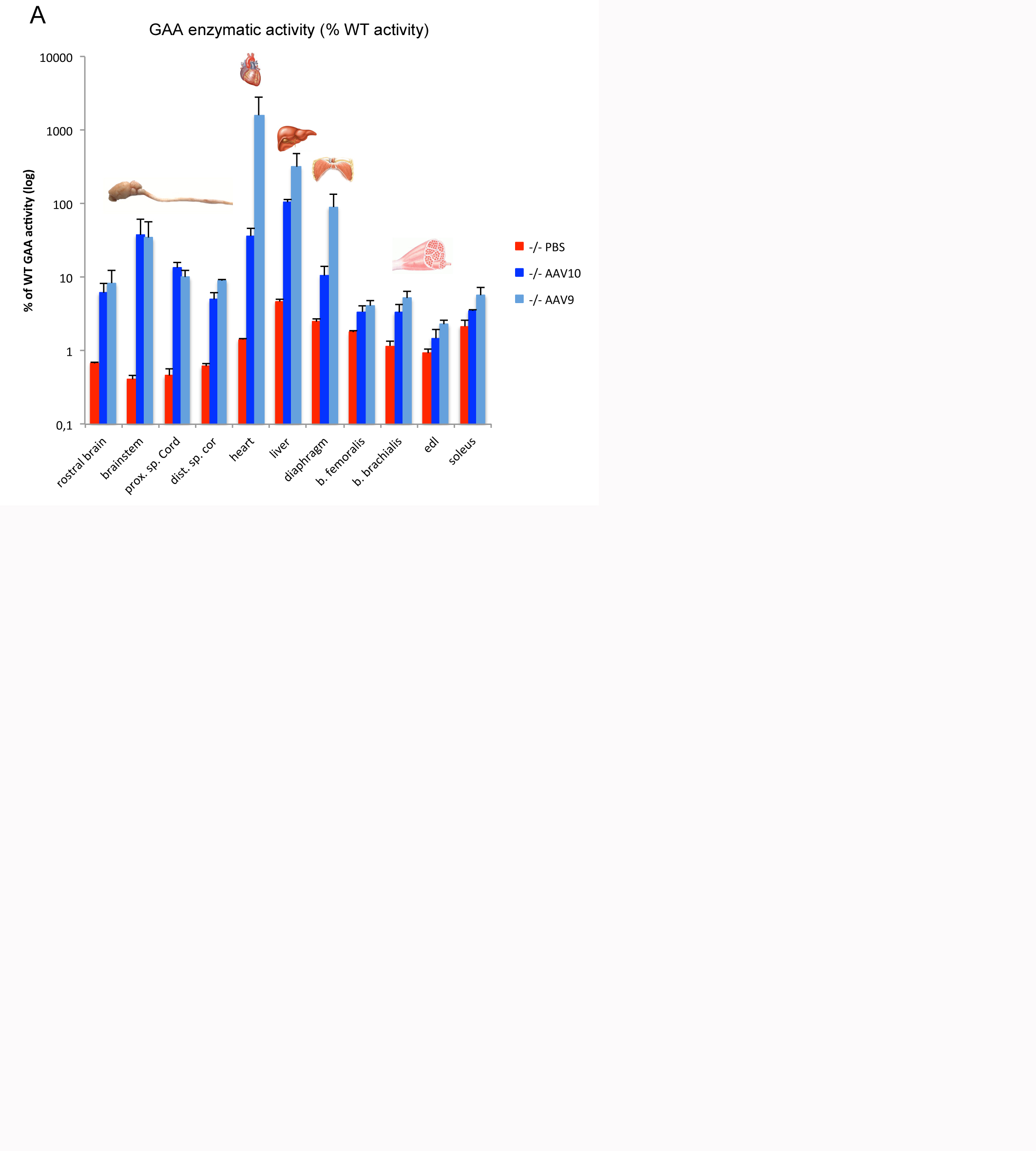
**

**Fig. S6.** GAA enzymatic activity in tissue extracts from the CNS, heart, liver, and muscles collected at 12 months

Samples were snap-frozen in liquid nitrogen rapidly after euthanasia and tissue extracts were assayed for GAA enzymatic activity using 4-methylumbelliferyl-α-D-glucopyranoside as substrate. Results are presented as % of the mean activity obtained in the WT animals (n=4 to 5 per group). Enzymatic activity is restored in the whole CNS, the heart, the liver, and the diaphragm. Less than 5% of the WT activity is measured in the muscles of the limbs.

**Fig. S7**

**
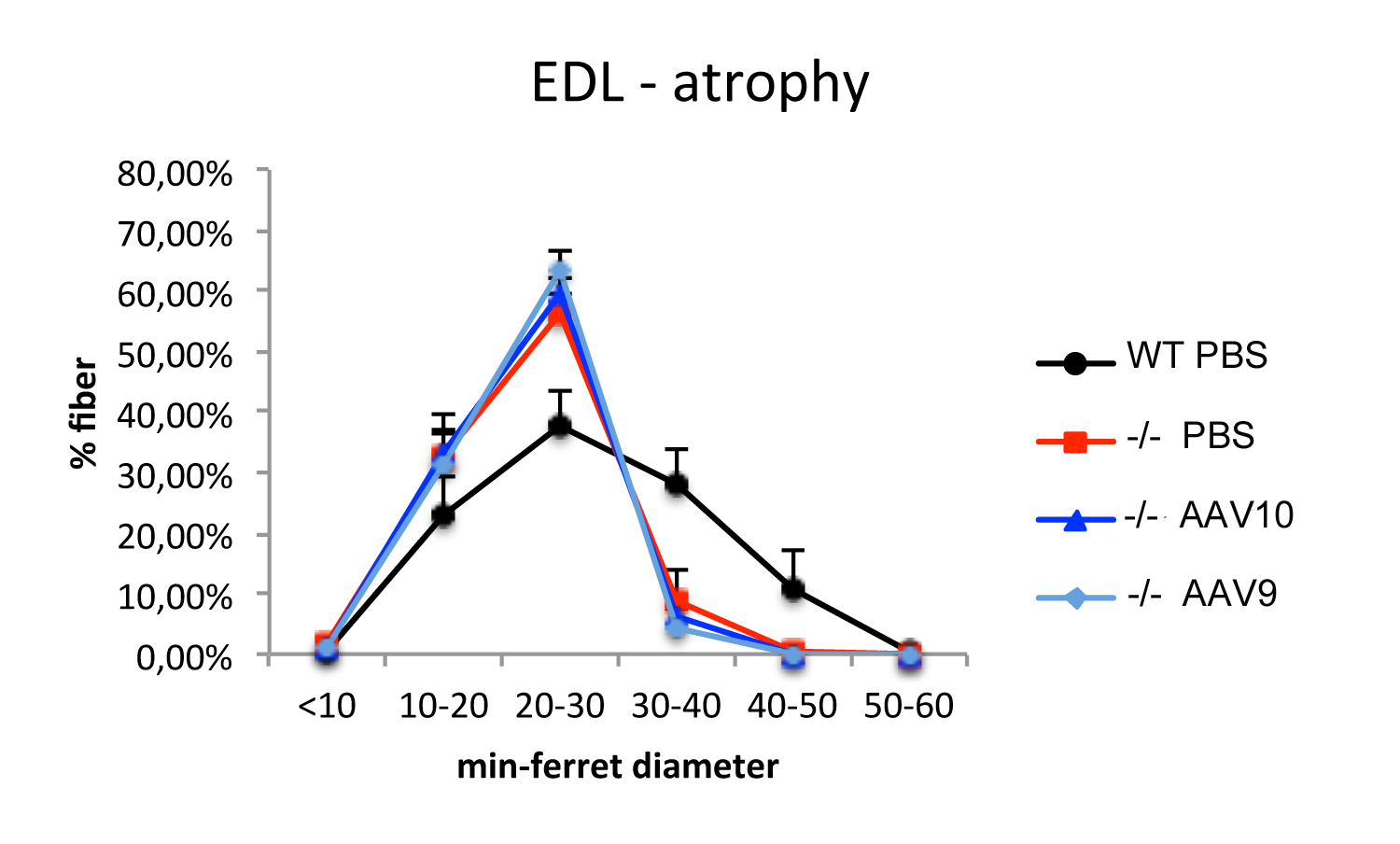
**

**Fig. S7** Myofiber minferret diameter frequency distribution in the *extensor digitorum longus* muscle

Myofibers in cross section were delineated using the NIS software and the diameters repartition was analyzed (n=4 animals per groups, n=200 myofibers per muscle). Muscles of Pompe mice have an increased proportion of small fibers showing atrophy. The treatments have no effect.

**Fig. S8**

**Fig. S8** Anti-GAA antibody titers measured by indirect ELISA at 12 months

rGAA (Myozyme) was coated on 96-wells plates and serial dilutions of sera were tested and compared to positive controls (rGAA intraperitoneally immunized mice) and to negative controls (sera of the animals collected prior to the injection). A cut-off value of 0.2 for optic density measurement was determined with the negative controls. Means are compared with bilateral Mann-Whitney test (AAVrh10 n=8 and AAV9 n=9).

**Fig. S9**


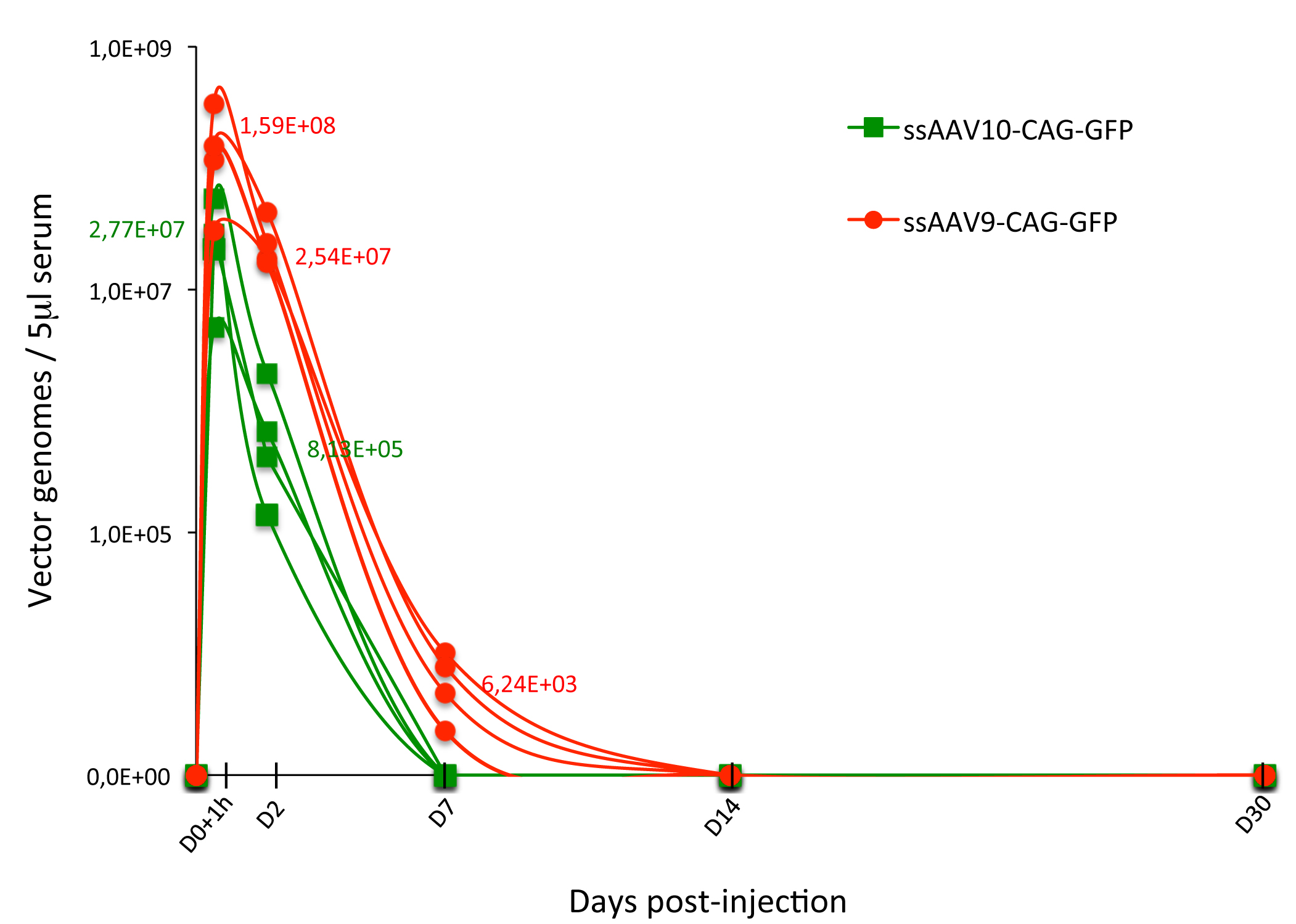


**Fig. S9** Viral particles distribution in the systemic circulation after administration in the CSF of the *cisterna magna*

Adult WT mice were injected with 10^11^ vg of AAVrh10-CAG-GFP or AAV9-CAG-GFP in 10 μl. Sera were collected and assayed for the presence of viral particles (polyA SV40 qPCR) 1 hour, 2 days, 7 days, 14 days, and 30 days after the intrathecal administration. Particles are detected in the blood at one hour (2 % ±0.7 of the injected AAVrh10 particles and 11.8 % ±4 of the injected AAV9 particles). Seven days after the injection, AAV9 particles are still quantifiable whereas AAVrh10 is no longer detectable (sensitivity of the assay 2x10^3^ vg per 5 μl of serum).

**Table S1** Causes of natural death during the long-term twelve-months study

| **Mice** | **Genotype** | **Treatment** | **Age of death (weeks)** | **Necropsy finding** | **Conclusion** |
| --- | --- | --- | --- | --- | --- |
| 16P1.42 | -/- | AAV10 | 20 | Hypertrophic cardiomyopathy | Heart failure |
| 17P1.1 | -/- | AAV10 | 20 | Hypertrophic cardiomyopathy | Heart failure |
| QP3.10 | WT | PBS | 33 | Salivary gland and sub-mandibular lymph node botryomycosis | Staphylococcal infection |
| 12P2.11 | -/- | PBS | 42 | Hypertrophic cardiomyopathy  Global muscular atrophy  Chronic cystitis with urinary stones | Heart failure |
| 13P4.5 | -/- | AAV9 | 40 | Hypertrophic cardiomyopathy  Global muscular atrophy  Chronic cystitis with urinary stones | Heart failure |
| 16P2.11 | -/- | PBS | 34 | Hypertrophic cardiomyopathy  Global muscular atrophy | Heart failure |
| 16P2.13 | -/- | AAV10 | 39 | Hypertrophic cardiomyopathy  Global muscular atrophy  Chronic cystitis with urinary stones | Heart failure |
| 16P2.15 | -/- | AAV10 | 40 | Hypertrophic cardiomyopathy  Global muscular atrophy  Chronic cystitis with urinary stones and urethral obstruction | Heart and kidney failure |
| 13P5.10 | -/- | AAV9 | 52 | Global muscular atrophy  Chronic cystitis with urinary stones and urethral obstruction | Kidney failure |
| 12P3.21 | -/- | AAV9 | 37 | Global muscular atrophy  Chronic cystitis with urinary stones and urethral obstruction | Kidney failure |

**Table S2** GAA activity restoration and glycogen storage correction in the CNS and heart 12 months after intrathecal AAV-CAG-hGAA therapy, individual data

| **Treatment** | **Tissue** | **GAA activity (nmol/h/mg prot)** | **GAA activity (% WT)** | **Glycogen correction**  **(% reduction)** |
| --- | --- | --- | --- | --- |
| AAVrh10 | Rostral brain | 1.33 | 4.7 | 88.6 |
|  |  | 0.95 | 3.3 | 70.1 |
|  |  | 3.64 | 12.7 | 100 |
|  |  | 1.10 | 3.8 | 78.6 |
|  | Brainstem | 5.69 | 6.1 | 100 |
|  |  | 3.85 | 4.1 | 36.8 |
|  |  | 97.79 | 105.3 | 95.3 |
|  |  | 32.81 | 35.3 | 100 |
|  | Proximal spinal cord | 13.03 | 16.9 | 89.8 |
|  |  | 6.61 | 8.6 | 100 |
|  |  | 13.37 | 17.4 | 77.8 |
|  |  | 8.69 | 11.3 | 100 |
|  | Distal spinal cord | 2.06 | 3.7 | 93.4 |
|  |  | 1.38 | 2.4 | 47.1 |
|  |  | 4.48 | 7.9 | 94.7 |
|  |  | 3.26 | 5.8 | 89.2 |
|  | Biceps femoris | 0.52 | 3.04 | 48,23 |
|  |  | 0.93 | 5.45 | 24.02 |
|  |  | 0.39 | 2.28 | 0 |
|  |  | 0.44 | 2.58 | 6,02 |
|  | Heart | 2.09 | 15.6 | 0 |
|  |  | 3.56 | 26.6 | 0 |
|  |  | 8.19 | 61.3 | 0 |
|  |  | 5.61 | 42 | 99.1 |

| **Treatment**  AAV9 | **Tissue** | **GAA activity (nmol/h/mg prot)** | **GAA activity (% WT)** | **Glycogen correction**  **(% reduction)** |
| --- | --- | --- | --- | --- |
|  | Rostral brain | 3.72 | 13 | 99.1 |
|  |  | 4.85 | 16.9 | 100 |
|  |  | 0.56 | 1.9 | 76.8 |
|  |  | 0.40 | 1.4 | 54.3 |
|  | Brainstem | 93.19 | 100.4 | 100 |
|  |  | 20.67 | 22.3 | 100 |
|  |  | 5.75 | 6.2 | 49.2 |
|  |  | 6.64 | 7.2 | 77.3 |
|  | Proximal spinal cord | 5.62 | 7.3 | 100 |
|  |  | 6.30 | 8.2 | 94.9 |
|  |  | 5.73 | 7.4 | 88.8 |
|  |  | 12.83 | 16.7 | 71.8 |
|  | Distal spinal cord | 4.92 | 8.7 | 86.8 |
|  |  | 5.04 | 8.9 | 85.9 |
|  |  | 5.77 | 10.2 | 87.5 |
|  |  | 4..52 | 8 | 52.9 |
|  | Biceps femoral | 0.37 | 2.18 | 0 |
|  |  | 0.68 | 4.02 | 33,70 |
|  |  | 0.78 | 4.61 | 10,73 |
|  |  | 0.95 | 5.58 | 0 |
|  | Heart | 29.93 | 223.9 | 41 |
|  |  | 21.28 | 159.2 | 0 |
|  |  | 721.76 | 5400.2 | 64 |
|  |  | 82.24 | 615.3 | 100 |
